# Two homologs of the Cat8 transcription factor are involved in the regulation of ethanol utilization in *Komagataella phaffii*

**DOI:** 10.1007/s00294-021-01165-4

**Published:** 2021-03-16

**Authors:** Diane Barbay, Monika Mačáková, Leander Sützl, Sonakshi De, Diethard Mattanovich, Brigitte Gasser

**Affiliations:** 1 Austrian Centre of Industrial Biotechnology (ACIB), Vienna, Austria; 2 Department of Biotechnology, Institute of Microbiology and Microbial Biotechnology, University of Natural Resources and Life Sciences (BOKU), Vienna, Austria; 3 Department of Food Technology, University of Natural Resources and Life Sciences (BOKU), Vienna, Austria

**Keywords:** *Komagataella phaffii*, *Pichia pastoris*, Yeast, Cat8, Sip4, CSRE, Transcription factor, Ethanol utilization, Carbon source

## Abstract

The transcription factors Cat8 and Sip4 were described in *Saccharomyces cerevisiae* and *Kluyveromyces lactis* to have very similar DNA binding domains and to be necessary for derepression of a variety of genes under non-fermentative growth conditions via binding to the carbon source responsive elements (CSREs). The methylotrophic yeast *Komagataella phaffii* (syn *Pichia pastoris*) has two transcription factors (TFs), which are putative homologs of Cat8 based on sequence similarity, termed Cat8-1 and Cat8-2. It is yet unclear in which cellular processes they are involved and if one of them is actually the homolog of Sip4. To study the roles of the Cat8 homologs in *K. phaffii*, overexpression or deletion strains were generated for the two TFs. The ability of these mutant strains to grow on different carbon sources was tested, and transcript levels of selected genes from the carbon metabolism were quantified. Our experiments showed that the TFs are required for the growth of *K. phaffii* on C2 carbon sources, but not on glucose, glycerol or methanol. *K. phaffii* deleted for Cat8-1 showed impaired growth on acetate, while both Cat8-1 and Cat8-2 are involved in the growth of *K. phaffii* on ethanol. Correspondingly, both TFs are participating in the activation of *ADH2*, *ALD4* and *ACS1*, three genes encoding enzymes important for the assimilation of ethanol. Different from *S. cerevisiae* and *K. lactis*, Cat8-1 is not regulating the transcription of the putative Sip4-family member Cat8-2 in *K. phaffii*. Furthermore, Cat8-1 is necessary for the activation of genes from the glyoxylate cycle, whereas Cat8-2 is necessary for the activation of genes from the carnitine shuttle. Neither Cat8-1 nor Cat8-2 are required for the activation of gluconeogenesis genes. Finally, the *CAT8-2* gene is repressed by the Mig1-2 transcription factor on glucose and autorepressed by the Cat8-2 protein on all tested carbon sources. Our study identified the involvement of *K. phaffii* Cat8-1 and Cat8-2 in C2-metabolism, and highlighted similarities and differences to their homologs in other yeast species.

**Supplementary Information:**

The online version contains supplementary material available at 10.1007/s00294-021-01165-4.

## Introduction

The yeast *Komagataella phaffii* (*Pichia pastoris*) adapts to different growth conditions through various mechanisms, including reprogramming of gene expression and protein synthesis (Hartner and Glieder 2006; Lin-Cereghino et al. 2006; Prielhofer et al. 2015). The release from glucose and catabolite repression alters the transcription of genes involved in numerous cellular processes, such as glycolysis, gluconeogenesis, tricarboxylic acid (TCA) cycle, and metabolism of alternative carbon sources (Prielhofer et al. 2015). Growth on gluconeogenic carbon sources such as glycerol and ethanol requires enzymes from gluconeogenesis and the glyoxylate cycle, among others. In the yeasts *Saccharomyces cerevisiae* and *Kluyveromyces lactis*, the genes encoding these enzymes are activated through upstream activation sites (UAS) found in their promoters, such as the carbon source responsive elements (CSREs) (Mehlgarten et al. 2015; Turcotte et al. 2010). The CSREs are under the control of two transcriptional regulators, which are members of the binuclear zinc cluster family: Cat8 and Sip4 (Roth et al. 2004; Vincent and Carlson 1998).

Cat8 (CATabolite repression) and Sip4 (Snf1 interacting protein) possess a highly similar N-terminal zinc cluster (Zn(II)_2_Cys_6_) binding domain (Rahner et al. 1996), but they share only little similarity in the rest of their protein sequences (Mehlgarten et al. 2015; Turcotte et al. 2010). Although both Cat8 and Sip4 were shown to bind the CSRE (consensus sequence: YCCRTTNRNCGG) (Roth et al. 2004; Vincent and Carlson 1998), Sip4 recognizes and binds to a more specific CSRE motif than Cat8, which probably explains why Cat8 and Sip4 contribute unequally to gene activation via binding to this motif (Hiesinger et al. 2001). In *S. cerevisiae* and *K. lactis*, Cat8 and Sip4 were described to be activators of transcription, but their mechanism of action in these two yeast are slightly different (Mehlgarten et al. 2015).

In *S. cerevisiae*, the expression and activities of *CAT8* (*Sc*Cat8) and *SIP4* (*Sc*Sip4) were shown to be regulated by glucose, in a process mediated by the Snf1 kinase (Hardie et al. 1998). This kinase has a fundamental role in glucose derepression through the activation of various transcriptional activators and the deactivation of the Cys_2_His_2_ zinc finger protein Mig1. In the presence of glucose, the transcription factor (TF) Mig1 binds to the promoter of genes such as *CAT8*, which represses their expression. On derepressing conditions, the Snf1 kinase phosphorylates Mig1, which leads to its inactivation and the consequent induction of *CAT8* transcription (Carlson 1999; Schüller 2003). The Cat8 protein is then activated via phosphorylation by Snf1, and induces the transcription of various genes involved in the growth on non-fermentable carbon sources such as genes of the C2 anabolism, the glyoxylate cycle, and gluconeogenesis (De Vit et al. 1997; Haurie et al. 2001; Hedges et al. 1995; Lesage et al. 1996; Randez-Gil et al. 1997; Tachibana et al. 2005). *Sccat8* knock-out mutants are unable to grow on gluconeogenic carbon sources such as glycerol, ethanol, lactate, and acetate (Hedges et al. 1995; Rahner et al. 1996), whereas *Scsip4* knock-out mutants have no apparent growth phenotype on any of the tested carbon sources. This indicates that in *S. cerevisiae*, Sip4 plays a minor role in CSRE-dependent regulation (Lesage et al. 1996). In addition, the *SIP4* promoter contains CSRE motifs which were shown to be bound by Cat8, indicating that Cat8 is regulating the transcription of *SIP4* in *S. cerevisiae* (Vincent and Carlson, 1998).

In the yeast *K. lactis*, both Cat8 (*Kl*Cat8) and Sip4 (*Kl*Sip4) are present but the regulatory networks are different than in *S. cerevisiae*. The *Klcat8* knock-out mutants can grow on glycerol but not on C2 carbon sources such as acetate and ethanol, which shows that unlike in *S. cerevisiae*, the gluconeogenesis encoding genes are not regulated by Cat8 nor Sip4 in *K. lactis* (Georis et al. 2000). On the other hand, contrary to *S. cerevisiae* where Cat8 but not Sip4 is required for growth on ethanol, both *Klcat8* and *Klsip4* knock-out mutants exhibit a growth defect on C2 carbon sources (Mehlgarten et al. 2015). It was also shown that only *Kl*Sip4 binds to the CSRE motifs in the promoters of the glyoxylate pathway genes and the carnitine shuttle encoding genes (Mehlgarten et al. 2015; Rodicio et al. 2008). In *K. lactis*, as in *S. cerevisiae*, Cat8 was shown to activate the transcription of Sip4. *Kl*Cat8 was also shown to be regulated via phosphorylation of a conserved serine residue (Ser-661) by the Snf1 kinase (Charbon et al. 2004).

Homologs of the Cat8 transcription factor were also found in other yeasts, although their function was not studied in as much depth as in *S. cerevisiae* or *K. lactis*. *Candida albicans* Cat8 (*Ca*Cat8) knock-out mutants have a similar phenotype to the wild-type in terms of gluconeogenesis, glyoxylate shunt, and ethanol utilization pathway, and *Ca*Cat8 does not seem to regulate the gluconeogenic gene *PCK1* nor *ICL1* (encoding isocitrate lyase, an enzyme of the glyoxylate cycle) (Ramirez and Lorenz 2009). Cat8 knock-out mutants in *Ogataea (Hansenula) polymorpha* exhibit a growth defect on glycerol, ethanol, and xylose, and have a higher ethanol production from xylose fermentation (Ruchala et al. 2017). Finally, in *Pichia guillermondii*, knocking-out the *CAT8* gene triggers respiro-fermentative metabolism of this Crabtree-negative yeast (Qi et al. 2014).

In *Komagataella phaffii*, two putative homologs of Cat8 termed *CAT8-1* (CBS7435 locus name PP7435_Chr2-0516) and *CAT8-2* (PP7435_Chr4-0434) are found. The *CAT8-2* gene was shown to be induced on limiting glucose (about 39-fold up-regulated compared to excess glucose) and on methanol (about sevenfold up-regulated compared to excess glucose), indicating that it is subject to glucose repression. No up- or down-regulation of *CAT8-1* was observed in the same conditions (Prielhofer et al. 2015). It is yet unclear which genes are regulated by the TFs Cat8-1 and Cat8-2 and if one of them is the homolog of Sip4. In order to study the role of *CAT8-1* and *CAT8-2* in *K. phaffii*, overexpression and knock-out mutants of each of these two TF genes were generated and the ability of the TF mutant strains to grow on different nutrient sources were tested. Based on the findings, the transcript levels of some selected genes from the carbon metabolism were quantified to see how their regulation patterns on different carbon sources were affected in the absence of either or both Cat8 homologs.

## Materials and methods

### Strains, primers and plasmids

All the *Komagataella phaffii* strains used in this study (Table 1) were derived from the wild-type strain CBS7435. *Escherichia coli* DH10B (Invitrogen) were used for cloning experiments.

**Table 1:** List of *K. phaffii* strains used in this study

Name	Target (ORF name)	Target (short name)	Target type (OE/KO/Prom)	Source
*cat8-2Δ*	PP7435_Chr4-0434	*CAT8-2*	KO	This study
*cat8-1Δ*	PP7435_Chr2-0516	*CAT8-1*	KO	This study
CAT8-2_OE	PP7435_Chr4-0434	*CAT8-2*	OE	This study
CAT8-1_OE	PP7435_Chr2-0516	*CAT8-1*	OE	This study
*cat8-1Δcat8-2Δ (CAT8dKO)*	PP7435_Chr2-0516 / PP7435_Chr4-0434	*CAT8-1/CAT8-2*	KO	This study
CAT8-1_HAtag	PP7435_Chr2-0516	*CAT8-1*	TAG	This study
CAT8-2_HAtag	PP7435_Chr4-0434	*CAT8-2*	TAG	This study
pCAT8-2_eGFP_CAT8-1KO	PP7435_Chr4-0434	*CAT8-2*	Prom	This study
pCAT8-2_eGFP_CAT8-2KO	PP7435_Chr4-0434	*CAT8-2*	Prom	This study
pCAT8-2_eGFP_WT	PP7435_Chr4-0434	*CAT8-2*	Prom	This study
pCAT8-2_eGFP_CAT8dKO	PP7435_Chr4-0434	*CAT8-2*	Prom	This study
pCAT8-1_eGFP_WT	PP7435_Chr2-0516	*CAT8-1*	Prom	This study
pCAT8-1_eGFP_CAT8-1KO	PP7435_Chr2-0516	*CAT8-1*	Prom	This study
pCAT8-1_eGFP_CAT8-2KO	PP7435_Chr2-0516	*CAT8-1*	Prom	This study
pCAT8-1_eGFP_CAT8dKO	PP7435_Chr2-0516	*CAT8-1*	Prom	This study
*snf1-2Δ*	PP7435_Chr1-0450	*SNF1-2*	KO	This study
*ssn3Δ*	PP7435_Chr1-1091	*SSN3*	KO	This study
*mig1-1Δ*	PP7435_Chr4-0661	*MIG1-1*	KO	Ata et al. (2017)
MIG1-1_OE	PP7435_Chr4-0661	*MIG1-1*	OE	Ata et al. (2017)
*mig1-2Δ*	PP7435_Chr1-1325	MIG1-2	KO	Ata et al. (2017)
MIG1-2_OE	PP7435_Chr1-1325	MIG1-2	OE	Ata et al. (2017)

### Sequence analyses, alignment and phylogenetic tree

Eight individual protein-BLAST searches were conducted on the ‘Non-redundant protein sequences (nr)’ database of NCBI using the following 6 functionally characterized protein sequences: CBF88979.1, Cat8 of *Aspergillus nidulans* (Todd et al. 1997, 1998); XP_018209149.1, Cat8 of *Ogataea polymorpha* (Ruchala et al. 2017); XP_453133.1/AAC23607.1, Cat8 of *K. lactis* (Georis et al. 2000); CAE00852.1, Sip4 of *K. lactis* (Mehlgarten et al. 2015); CAA55139.1, Cat8 of *S. cerevisiae* (Hedges et al. 1995) and CAA89382.1, Sip4 of *S. cerevisiae* (Lesage et al. 1996) along with XP_002491690.1 and XP_002493979.1, Cat8-1 and Cat8-2 of *K. phaffii*, given here with their NCBI identifiers. For the two *K. phaffii* proteins, the entries of the reference sequence of *K. phaffii* GS115 were taken, which are identical to the protein sequences in *K. phaffii* CBS7435 used for the experiments in this study. The search was restricted to Saccharomycetes (Yeast; taxid: 4891) and a maximum *E*-value of 9e−30.

All individual BLAST search results were combined and protein ID duplicates were removed (361 sequences). Then, all sequences containing invalid protein characters (B J O U X Z) were removed from the selection (352 sequences), remaining sequences were filtered for a minimum length of 600 amino acids (275 sequences) and sequences in the selection of 99% sequence identity or higher were represented in the selection by only one sequence of that cluster to reduce sequence redundancy (157 sequences). Together with all characterized Cat8 and Sip4 sequences this resulted in a selection of 161 sequences.

The selection was aligned with MAFFT G-INS-I (Katoh and Standley 2013) and renamed with SeqScrub (Foley et al. 2019) according to taxonomy. The alignment was trimmed for positions with > 90% gaps by trimAl (Capella-Gutierrez et al. 2009) and a maximum likelihood tree was calculated with PhyML (Guindon et al. 2010) using the LG amino acid substitution model, the best of NNI and SPRs moves to optimize tree topology and SH-like branch support. The tree was rooted on midpoint. All protein identifiers (NCBI accession numbers) of sequences used in the alignment are listed in Supplementary material 1.

### Prediction of transcription factor binding sites (TFBS)

The regions upstream of all *K. phaffii* genes (1000 bps upstream of the start codon) were analyzed for the occurrence of the CSRE by MatInspector (matrix F$CSRE). Comparative analysis of TFBS in *S. cerevisiae*, *K. lactis* and *K. phaffii* promoters was done by Yeastract+ (http://www.yeasttract-plus.org; Monteiro et al. 2020).

### Media

YPD medium contained 10 g/L yeast extract, 20 g/L soy peptone and 2% glucose as carbon source. YPD agar plates consisted of 10 g/L yeast extract, 20 g/L soy peptone, 2% glucose as carbon source and 20 g/L agar–agar. The YPD liquid medium and the YPD agar plates were supplemented with the appropriate antibiotics (zeocin 50 μg/mL, geneticin 500 μg/mL, nourseothricin 100 μg/mL) when needed.

LB media consisted of 10 g/L soy peptone, 5 g/L yeast extract, 5 g/L NaCl. 20 g/L agar–agar was added to prepare LB agar plates.

ASMv6 medium contained per liter 6.3 g (NH_4_)_2_HPO_4_, 0.8 g (NH_4_)_2_SO_4_, 0.49 g MgSO_4_ · 7H_2_O, 2.64 g KCl, 0.0535 g CaCl_2_ · 2H_2_O, 22 g citric acid monohydrate, 1.47 mL PTM, 2 mL biotin (0.2 g/L), 20 mL NH_4_OH (25%) with additional carbon source according to the purpose. For limited glucose condition 25% m2p kit Polysaccharide and 0.078% enzyme was used (m2p-labs GmbH, Germany).

PTM0 stock solution contained per liter 0.08 g NaI, 6.0 g CuSO_4_ · 5H_2_O, 3.36 g MnSO_4_ · H_2_O, 0.2 g Na_2_MoO_4_ · 2H_2_O, 0.82 g CoCl_2_, 0.02 g H_3_BO_3_, 20.0 g ZnCl_2_, 65.0 g FeSO_4_ · 7H_2_O and 5.0 g mL H_2_SO_4_ (95–98%).

YNB without thiamine consisted of 10 g/L (NH_4_)_2_SO_4_, 0.2 g/L biotin, 0.8 mg/L Ca-pantothenate, 0.004 mg/L folic acid, 4 mg/L inositol, 0.8 mg/L niacin, 0.4 mg/L p-aminobenzoic acid, 0.8 mg/L pyridoxine HCl, 0.4 mg/L riboflavin, 1 mg/L H_3_BO_3_, 0.08 mg/L CuSO_4_, 0.2 mg/L KI, 0.4 mg/L FeCl_3_, 0.8 mg/L MnSO_4_ · H_2_O, 0.4 mg/L Na_2_MoO_4_ · 2H_2_O, 0.8 mg/L ZnSO_4_, 2 g/L KH_2_PO_4_, 1 g/L MgSO_4_, 200 mg/L NaCl, 200 mg/L CaCl_2_ with additional carbon source according to the purpose. For the limiting glucose condition 25% m2p kit Polysaccharide and 0.078% enzyme was used (m2p-labs GmbH, Germany).

Phosphate buffered saline (PBS) consisted of 1.8 g/L Na_2_HPO_4_⋅2H_2_O, 0.24 g/L KH_2_PO_4_, 8 g/L NaCl, 0.2 g/L KCl.

### Genomic DNA extraction and PCR

Genomic DNA was extracted from overnight cultures using the Wizard genomic DNA purification kit (Promega Corp., USA) according to the protocol of the manufacturer. All PCRs were performed using the Q5 polymerase (New England Biolabs, Inc., USA) following the recommendations of the manufacturer or using the OneTaq 2× master mix with GC buffer (New England Biolabs, Inc., USA). The PCRs were used to amplify DNA for cloning and for verifying positive transformants.

### Construction of overexpression and knock-out strains

The *K. phaffii* CBS7435 chromosomal regions of the selected transcription factors are: PP7435_Chr2-0516 for *CAT8-1* and PP7435_Chr4-0434 for *CAT8-2*. The sequences of these genes were retrieved from http://pichiagenome-ext.boku.ac.at.

Golden Gate Assembly (GGA; Engler et al. 2008) was used for the construction of the overexpression and knock-out cassettes using the Golden*Pi*CS vector series (Prielhofer et al. 2017). Internal *Bsa*I or *Bpi*I sites within the CDS or homologous regions for the integration of knock-out cassettes were eliminated by designing primers which enabled to overlap the modified regions by PCR or ordering in vitro synthesized gBlocks where these nucleotides were mutated without altering the originally encoded amino acids.

#### Overexpression

For overexpression cassettes, the CDS of the corresponding genes were amplified from the *K. phaffii* CBS7435 genome by PCR and cloned into a plasmid carrying the KanMX marker cassette and a region for homologous integration into the *AOX1* terminator. The *THI11* promoter, the promoter of a gene which encodes a protein involved in the synthesis of the thiamine precursor hydroxymethyl pyrimidine (HMP), was used for the overexpression of selected TFs (Delic et al. 2013). The expression capacity of this promoter can be controlled by the presence or absence of thiamine (Landes et al. 2016). For all overexpression cassettes, the *RPS3tt* transcription terminator was used. Primers used for the generation of overexpression cassettes are given in Supplementary table S1. The transformants were verified by colony PCR and gene copy number determination (positive clones had two copies of the gene of interest: the native one and the one of the overexpression cassette).

#### Knock-outs

*Komagataella phaffii* knock‐out strains were constructed by using CRISPR/Cas9-based homology-directed genome editing (Gassler et al. 2019). The homologous regions (HR) were amplified by PCR from the *K. phaffii* CBS7435 genome. These homologous regions were selected from upstream (5′) and downstream (3′) of the target gene with an approximate 1000 bp length. The primers used for amplification of these homologous fragments and elimination of internal *Bsa*I sites are given in Supplementary table S2. The flanking upstream and downstream homologous regions of the target gene were assembled with each other in a plasmid by GGA.

A single guide RNA was designed and amplified based on a protospacer adjacent motif (PAM) sequence identified in 50–200 bp upstream of the CDS of the selected TFs [see Gassler et al. (2019) for more details on the construction of the single guide RNA]. The guide RNA was cloned under the control of the *GAP* promoter and the *RPS25A*tt terminator into a plasmid containing the humanized Cas9 CDS under the control of P_LAT1_ or P_PFK300_ and the *ScCYC*tt terminator by GGA. For the integration of the knock-out cassettes, the plasmids carrying the fused homologous regions were used as templates to amplify the fragments by PCR. 3–5 µg of amplified homologous DNA and 0.5–1 µg of circular CRISPR/Cas9 plasmid DNA were simultaneously transformed into *K. phaffii* CBS7435 by electroporation (Gasser et al. 2013). The knock-out (KO) strains were checked by two PCRs using (1) primers binding in the genome outside of the targeted deletion sites and (2) binding in the CDS of the targeted TFs. After confirmation of the TF deletions, true KO transformants were passaged at least three times on YPD to lose the CRISPR/Cas9 plasmid.

#### Transformation of *K. phaffii*

Prior to *K. phaffii* transformation by electroporation (Gasser et al. 2013), the overexpression plasmids were linearized within the genome integration locus by *Asc*I and purified (innuPREP DOUBLEpure Kit, Analytik Jena, Germany). PCR-amplified homologous regions of knock-out cassettes were directly purified without linearizing. *K. phaffii* transformation was performed by electroporation (BioRad Gene Pulser, 2000 V, 25 µF and 200 Ω) by using 0.5–1 µg of each linearized overexpression plasmid or 3–5 µg of purified knock-out fragments and 0.5–1 µg of circular CRISPR/Cas9 plasmid. Transformed cells were then regenerated by incubation at 30 °C for 1.5–3 h in YPD medium (280 rpm) and then plated on YPD plates including the appropriate antibiotics concentration (zeocin 50 μg/mL, geneticin 500 μg/mL, nourseothricin 100 μg/mL). After 48–72 h at 30 °C, randomly selected transformants were streaked on selective YPD plates and incubated 48 h at 30 °C.

### Gene copy number determination

Gene copy number (GCN) was determined by quantitative real-time PCR (qRT-PCR). Genomic DNA was extracted from overnight cultures using the Wizard genomic DNA purification kit (Promega Corp., USA). The GCN was determined by the relative quantification of the TF of interest sequence compared to wild-type *K. phaffii* CBS7435 (carrying a single native copy of the TF gene of interest). The amplifications were carried out using 4.5 µL of genomic DNA solution at a concentration of 1.777 ng/µL with 0.25 µL of both forward and reverse primers (final concentration: 10 µM) and 5 µL of 2× qPCR S’Green BlueMix (Biozym Scientific GmbH, Germany). Amplifications were done in a Rotor-Gene Q instrument (QIAGEN GmbH, Germany). The GCN in the *K. phaffii* mutant strains were calculated relative to the corresponding wild-type control using the threshold cycle (ΔΔ*CT*) method. All signals were normalized to *ACT1* (PP7435_Chr3-0993). The primers used for qRT-PCR analysis are provided in Supplementary table S3.

### Growth assays in liquid medium

The wild-type, overexpression and deletion strains were inoculated at OD_600_ 0.01 in 100 μL of YNB without thiamine containing either 2% glucose, 2% glycerol, 2% ethanol or 1% methanol in a 96-well sterile microtiter plate. Growth on acetate was assessed in 100 μL of YNB or YP containing 1% or 2% acetate. The plate was incubated in a TECAN Sunrise plate reader at 30 °C for 24–48 h (99 cycles, interval 14:39 min) with constant shaking (before measurement 5 s, inside, normal; between cycles 870 s, inside, normal). The absorbance at 600 nm in each well was measured every 15 min.

For each strain, three to four biological replicates were cultivated each in three wells. The blank value (OD_600_ of the media without cells) was subtracted from the raw OD values to obtain the corrected ODs. The corrected OD_600_ of the replicates for a given strain were then averaged. The average corrected OD_600_ was plotted against the time to obtain the growth curves. For the calculation of the specific growth rates, the average corrected OD_600_ was divided by the average initial OD_600_ and the natural logarithm was applied. The growth rate is given by the slope of the log-transformed ODs, the maximal growth rate being identified as the maximum value of the slope (Toussaint and Conconi 2006).

### RNA extraction and transcript levels analysis

The wild-type, overexpression and deletion strains were grown on ASMv6 medium with limiting glucose to OD_600_ 7.0–8.0, washed twice in PBS, inoculated at OD_600_ 3.5–4.5 in ASMv6 medium containing either 2% glucose, 2% glycerol, 2% ethanol or 1% methanol and grown for 5 h, to have an induction on glucose, glycerol, ethanol, and methanol, respectively. Samples were then collected by centrifugation at full speed at 4 °C, and cell pellets were resuspended in 1 mL TRI reagent solution (Invitrogen) and stored at −70 °C until further use. Cells were mechanically disrupted using 500 µL of glass beads in a ribolyzer (5.5 m/s for 40 s), and the total RNA extraction was performed according to the TRI reagent protocol. RNA concentrations and purity were analyzed with a Nanodrop spectrophotometer. DNAse treatment of isolated RNA samples was performed with a DNA-free kit (Invitrogen) and cDNA was synthesized using oligo(dT)_23_ primers (New England Biolabs, Inc., USA) and the Biozym cDNA synthesis kit according to directions of the manufacturer (Biozym Scientific GmbH, Germany). The Real-time PCR reactions were performed on a Rotor-Gene Q instrument (QIAGEN GmbH, Germany) using Blue S’Green qPCR Mix (Biozym Scientific GmbH, Germany) according to the manufacturer’s instructions. Changes in transcript levels in the *K. phaffii* mutant strains were calculated relative to the corresponding wild-type control using the threshold cycle (ΔΔ*CT*) method. All signals were normalized to the expression of the actin gene *ACT1* (PP7435_Chr3-0993). The primers used for qRT-PCR analysis are provided in Supplementary table S4.

### Construction of the HA-tagged Cat8-1 and Cat8-2 strains

Initially, the tagging of Cat8-1 and Cat8-2 was tested with a 3xFLAG tag at the native locus using the CRISPR/Cas9-based homology-directed genome editing (Gassler et al. 2019), but no clones were obtained after transformation. Another approach was transforming plasmids containing the coding sequences of *CAT8-1* and *CAT8-2* with a 3xFLAG tag before their STOP codons under the control of their native promoters into *K. phaffii cat8-1Δ* (for Cat8-1-3xFLAG) and *cat8-2Δ* (for Cat8-2-3xFLAG) deletion strains. Again no clones were obtained. Since the single deletion strains of *CAT8-1* and *CAT8-2* were viable, it seemed that introducing a FLAG-tagged copy of Cat8-1 and Cat8-2 was detrimental to the cells. Therefore tagging of Cat8-1 and Cat8-2 with the HA tag was tested, and was proven to be a successful strategy yielding viable transformants.

To construct the HA-tagged Cat8-1 and Cat8-2 strains, promoter regions (upstream 1000 bp regions), coding sequences, and terminator regions of *CAT8-1* and *CAT8-2* were amplified from *K. phaffii* CBS7435 genomic DNA by PCR. Reverse primers for the amplification on the coding sequences were designed so there is insertion of the HA tag (encoded by TACCCATACGATGTTCCAGATTACGC) before the STOP codons of the two genes. The PCR fragments were assembled by GGA in a vector carrying the *AOX1* terminator homologous regions (for integration into the *K. phaffii* genome) and the KanMX marker cassette. 0.5-1 µg of linearized plasmids were transformed into *K. phaffii cat8-1Δ* (for Cat8-1-HA) and *cat8-2Δ* (for Cat8-2-HA) deletion strains. Transformants were controlled by colony PCR and qRT-PCR (for gene copy number determination). The selected clones were also grown on YNB liquid medium supplemented with 1% ethanol to make sure that the insertion of the tagged version of the proteins in the deletion strains restored their growth on this carbon source.

### eGFP reporter assay

#### Cloning of eGFP under the control of P_CAT8-1_ and P_CAT8-2_

Promoter regions (upstream 1000 bp regions) of *CAT8-1* and *CAT8-2* were amplified from *K. phaffii* CBS7435 genomic DNA by PCR (Primers in Supplementary table S5). The respective promoters, the eGFP coding sequence and the *ScCYC*tt were assembled by GGA in a vector containing *AOX1* terminator homologous regions (for integration into *K. phaffii* genome) and the KanMX marker cassette. 0.5–1 µg of the linearized plasmids were transformed into *K. phaffii* CBS7435 (WT), *cat8-1Δ*, *cat8-2Δ*, and *cat8-1Δcat8-2Δ* deletion strains. Transformants were controlled by colony PCR and qRT-PCR for gene copy number determination.

#### Reporter assay

The wild-type and positive transformants of the wild-type, *cat8-1Δ*, *cat8-2Δ*, and *cat8-1Δcat8-2Δ* deletion strains carrying the eGFP coding sequence under the control of the respective promoters were grown on ASMv6 medium with limiting glucose (polysaccharide solution) at 25 °C to OD_600_ 7.0–8.0, washed twice in PBS and inoculated at OD_600_ 3.5–4.5 in ASMv6 medium containing either 2% glucose, 2% glycerol, 2% ethanol or 1% methanol and grown for 5 h at 25 °C, to have an induction on glucose, glycerol, ethanol, and methanol, respectively. After induction, cells were analyzed by flow cytometry.

#### Flow cytometry

Cells were diluted in PBS (KH_2_PO_4_ 0.24 g/L, Na_2_HPO_4_⋅2 H_2_O 1.8 g/L, KCl 0.2 g/L, NaCl 8 g/L) to OD_600_ 0.2. The forward and side scatter of 10,000 cells for each sample as well as their green fluorescence (FL1 channel, 505–545 nm) were then measured on a CytoFLEX flow cytometer (Beckman Coulter). The Kaluza analysis software (Beckman Coulter) was used to analyze the data. GFP-positive cells were gated using the WT_P_CAT8-1__eGFP and WT_P_CAT8-2__eGFP strains as reference.

## Results

### Phylogenetic analysis of Cat8 and Sip4 homologs in *Saccharomycetes*

The transcription factors Cat8 and Sip4 both belong to the family of binuclear zinc cluster proteins. These proteins are to date only found in fungi and typically possess a zinc cluster motif with the following consensus sequence: CysX_2_CysX_6_CysX_5–12_CysX_2_CysX_6–8_Cys. The six conserved cysteine residues are involved in the folding of the zinc cluster domain, which is important for DNA recognition. Within the DNA binding domain, a linker region connects the zinc cluster motif to a Leucine zipper-like dimerization domain, involved in protein–protein interactions. As usual in binuclear zinc cluster proteins, the DNA binding domain is located near the N-terminus, while the activation domain is found at the C-terminus (Todd and Andrianopoulos 1997).

Based on reciprocal BLAST analysis, *K. phaffii* Cat8-1 and Cat8-2 were found to be two homologs of *S. cerevisiae* Cat8 (Valli et al. 2016). Both Cat8-1 and Cat8-2 exhibit the characteristic binuclear zinc cluster Zn(II)_2_Cys_6_ binding domain located at the N-terminus. To find out if one of these two genes could encode a protein structurally related to Sip4, a phylogenic analysis was performed.

**Fig. 1:**
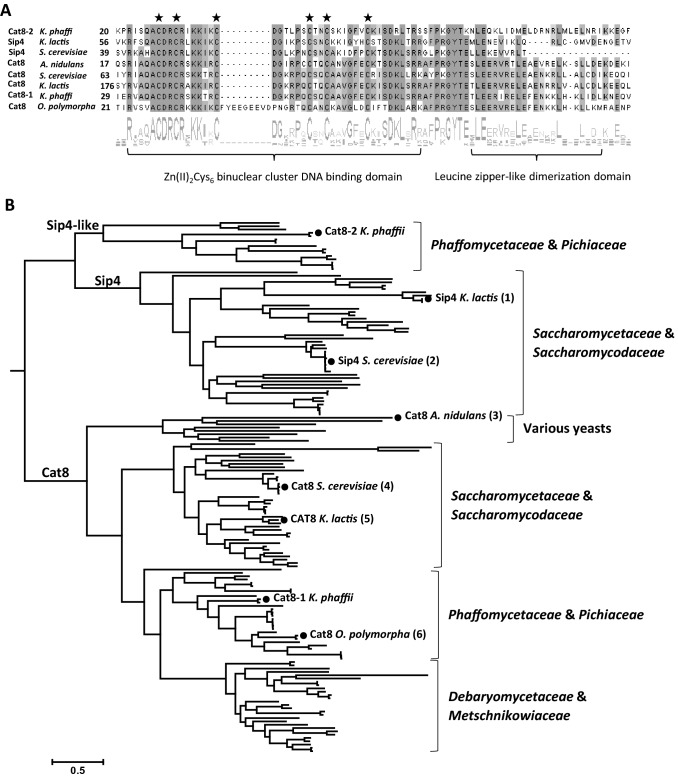
Phylogenetic and sequence analysis of sequence homologs of Cat8 and Sip4. **A** Multiple sequence alignment and sequence logo of the fungal Zn(II)_2_Cys_6_ DNA binding domain of the 6 functionally characterized Cat8 and Sip4 as well as *K. phaffii* Cat8-1 and Cat8-2. Identical residues are shown in grey boxes and the cysteine residues involved in forming the DNA binding domain are marked with a star. **B** Phylogenetic tree based on 161 full length amino acid sequences of Cat8 and Sip4 homologs in *Saccharomycetes* (Yeast; taxid:4891). Full circles (•) represent the positions of the 6 functionally characterized Cat8 and Sip4 as well as *K. phaffii* Cat8-1 and Cat8-2 protein sequences that were used for BLAST search: 1. Mehlgarten et al. 2015, 2. Lesage et al. 1996, 3. Todd et al. 1997, 4. Hedges et al. 1995, 5. Georis et al. 2000, 6. Ruchala et al. 2017

A total of 161 sequences from various yeasts including 6 functionally characterized Cat8 and Sip4 protein sequences from *S. cerevisiae* (Cat8 1433 aa, Sip4 829 aa), *K. lactis* (Cat8 1445 aa, Sip4 717 aa), *O. polymorpha* (Cat8 942 aa) and *A. nidulans* (FacB 867 aa) as well as *K. phaffii* Cat8-1 (1036 aa) and Cat8-2 (887 aa) were aligned, and their phylogenic tree was calculated (Fig. 1).

Generally, a high sequence variation was observed within the Cat8 and Sip4 variants from different yeasts. Only the DNA binding domain was conserved in all sequences. Specifically, the six cysteine residues involved in forming this DNA binding domain are well conserved among the Cat8 and Sip4 homologs (Fig. 1A). The adjacent Leucine zipper dimerization domain in the C-terminal region of the binuclear cluster is conserved as well (Fig. 1A). Apart from this, there is not much sequence similarity observed.

In the phylogenetic tree, we can observe a clear separation of Sip4 and Cat8 homologs and a further subseparation between *Saccharomycetaceae* and other yeast genera (Fig. 1B). Five major clades were identified according to functional annotation and fungal genus: the Cat8-2 clade of *Phaffomycetaceae* and *Pichiaceae* (containing *K. phaffii* Cat8-2), the Sip4 clade of *Saccharomycetaceae* and *Saccharomycodaceae* (containing the characterized Sip4 proteins from *S. cerevisiae* and *K. lactis*), the Cat8 clade of *Saccharomycetaceae* and *Saccharomycodaceae* (containing the characterized Cat8 proteins from *S. cerevisiae* and *K. lactis*), the Cat8 clade of *Phaffomycetaceae* and *Pichiaceae* (containing *K. phaffii* Cat8-1 and *O. polymorpha* Cat8), and finally the Cat8 clade of *Debaryomycetaceae* and *Metschnikowiaceae*. In addition, a small clade of 8 sequences of 5 different yeast genus and one *Aspergillus* Cat8 is also observed (Fig. 1B). A listing of species and sequences present in each clade is given in Supplementary material 1.

Based on this full-length protein sequences analysis, we can confirm that the Cat8-1 protein from *K. phaffii* clusters with other characterized Cat8 proteins. The Cat8-2 protein from *K. phaffii* on the other hand clusters more closely with Sip4 proteins, although constituting a different clade than the characterized Sip4 homologs of *S. cerevisiae* and *K. lactis* (Fig. 1B).

### *CAT8-1* and *CAT8-2* are essential for ethanol assimilation in *K. phaffii*

**Fig. 2:**
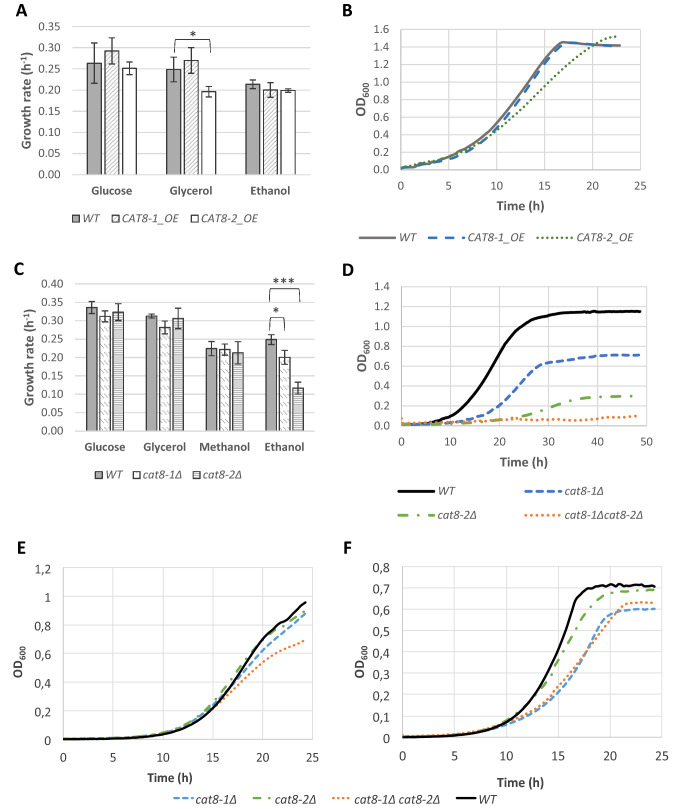
Influence of *CAT8-1* and *CAT8-2* overexpression and deletion on carbon source utilization. **A** Growth rates of the *CAT8-1* and *CAT8-2* overexpression mutants and the *K. phaffii* wild-type (WT) on YNB without thiamine with 2% glucose, 2% glycerol and 1% ethanol. **B** Growth curves of the *CAT8-1* and *CAT8-2* overexpression mutants and the WT on YNB without thiamine with 2% glycerol. **C** Growth rates of *cat8-1Δ*, *cat8-2Δ* and the WT on 2% glucose, 2% glycerol, 1% methanol and 1% ethanol. **D** Growth curves of *cat8-1Δ*, *cat8-2Δ*, the double knock-out *cat8-1Δ cat8-2Δ* and the WT on 1% ethanol. **E** Growth curves of *cat8-1Δ*, *cat8-2Δ*, the double knock-out *cat8-1Δ cat8-2Δ* and the WT on YP + 2% acetate. **F** Growth curves of *cat8-1Δ*, *cat8-2Δ*, the double knock-out *cat8-1Δ cat8-2Δ* and the WT on YNB + 2% acetate. Error bars represent the standard deviations of three to four independent biological samples each measured in technical triplicates. The statistically significant differences compared to the WT are indicated with asterisks (Student’s *t* test; *p < 0.05, **p < 0.01, ***p < 0.001)

To identify in which cellular processes the TFs Cat8-1 and Cat8-2 are involved, overexpression and knock-out strains of *CAT8-1* and *CAT8-2* were generated. For the overexpression, both genes were cloned under the control of the tunable *THI11* promoter, which is repressed in presence of thiamine in the growth medium and induced in thiamin-depleted conditions (Delic et al. 2013; Landes et al. 2016), and transformed in the wild-type *K. phaffii* strain CBS7435. The knock-out strains of *CAT8-1* and *CAT8-2* were generated using CRISPR/Cas9-based homology-directed genome editing (Gassler et al. 2019). The growth of the overexpression and knock-out strains, as well as the K. phaffii wild-type, was assessed in liquid cultures with YNB (without thiamine for the overexpression strains) containing either 2% glucose, 2% glycerol, 2% ethanol or 1% methanol (Fig. 2).

No significant difference was observed between the specific growth rates of the *CAT8-1* overexpression strains and the wild-type on the different carbon sources (Fig. 2A). For the *CAT8-2* overexpression strains, there was no significant difference to the growth rates of the wild-type on glucose and methanol. However, the growth rate of the *CAT8-2* overexpression strain was reduced on glycerol (Fig. 2A), and it reached the stationary phase later than the wild-type on this carbon source (Fig. 2B).

For the knock-out strains *cat8-1Δ* and *cat8-2Δ*, no significant differences in the specific growth rates were observed compared to the ones of the wild-type on glucose, glycerol, and methanol (Fig. 2C). However, *cat8-1Δ* and *cat8-2Δ* had significantly lower growth rates than the wild-type on ethanol (0.20 h^−1^ for *cat8-1Δ* and 0.12 h^−1^ for *cat8-2Δ* against 0.25 h^−1^ for the wild-type), and they reached a lower OD_600_ at the end of the culture (Fig. 2D). Furthermore, a *cat8-1Δcat8-2Δ* double knock-out was generated and it was shown to be completely unable to grow on ethanol (Fig. 2D). When a single copy of the *CAT8-1* or the *CAT8-2* genes under the control of their native promoters was introduced in the *cat8-1Δ* or *cat8-2Δ* mutant strains, respectively, the growth was again similar to that of the wild-type, showing that the phenotype observed on ethanol is caused solely by the lack of these two genes (Supplementary Figure S1). In contrast, complementation of *cat8-2Δ* or the double knock-out *cat8-1Δ cat8-2Δ* with *S. cerevisiae SIP4* (under control of the *CAT8-2* promoter) did not rescue growth on ethanol (not shown), indicating that *Sc*Sip4 was neither able to complement Cat8-1 nor Cat8-2 under the analyzed conditions. Therefore, it was concluded that Cat8-1 and Cat8-2 are two essential transcription factors for the growth of *K. phaffii* on ethanol.

#### Deletion of *CAT8-1*, but not of *CAT8-2*, impairs growth of *K. phaffii* on acetate

Growth of the knock-out strains *cat8-1Δ* and *cat8-2Δ*, and the *cat8-1Δcat8-2Δ* double knock-out was also assessed on acetate, another C2 carbon source. As it has been reported that *K. phaffii* deleted for another TF, Mxr1, only showed impaired growth on complex medium, but not YNB (Sahu and Rangarajan 2016), we cultivated the strains on both YP-A (Fig. 2E) and YNB-A (Fig. 2F), using two different acetate concentrations (1 and 2%) each. As can be seen in Fig. 2F, *cat8-1Δ* and the double knock-out *cat8-1Δcat8-2Δ* show impaired growth on acetate, which is more manifested on 2% acetate in YNB than in the other tested conditions. In YNB-A, growth rates were reduced to 77% in the *cat8-1Δ* mutant and to 70% in the double knock-out on both acetate concentrations. A slight delay in growth was also observed for *cat8-2Δ.*

These results are in contrast to *K. lactis*, where *Klsip4Δ* and the double knock-out *Klsip4Δcat8Δ* could not grow on acetate at all, and *Klcat8Δ* was growth impaired (Mehlgarten et al. 2015).

### *CAT8-1* and *CAT8-2* expression levels are higher on ethanol and methanol than on glucose and glycerol

As a next step, the transcript levels of *CAT8-1* and *CAT8-2* in the wild-type induced on different carbon sources were determined.

**Fig. 3:**
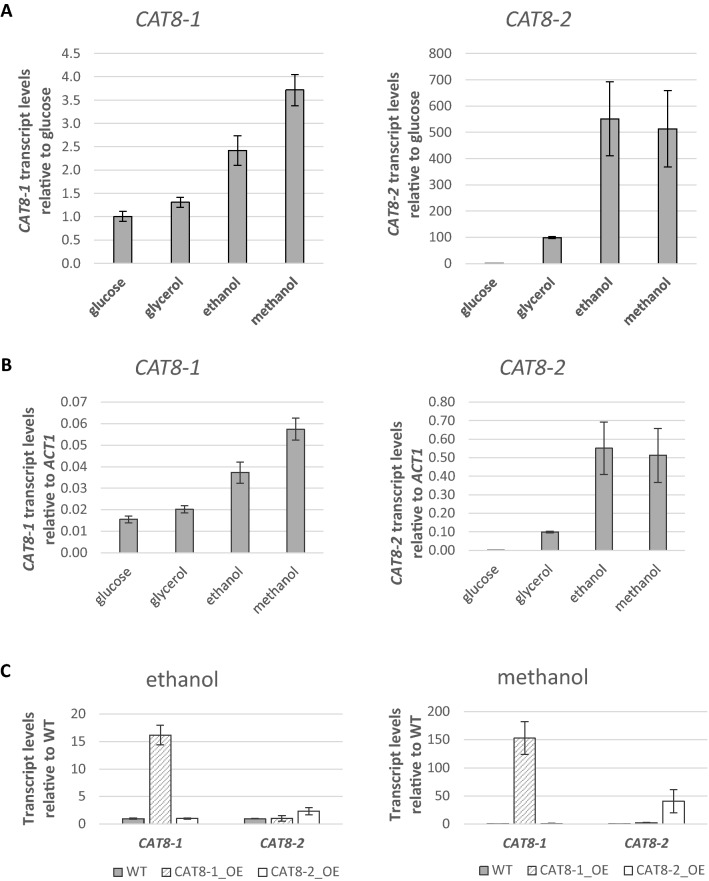
Influence of the carbon source on *CAT8-1* and *CAT8-2* gene expression. **A** Transcript levels of *CAT8-1* and *CAT8-2* in the *K. phaffii* wild-type CBS7435 induced on different carbon sources. Gene expression levels were normalized to the reference gene *ACT1* and quantified relative to the levels on glucose (set to 1.0) for each carbon source. **B** Expression strength of *CAT8-1* and *CAT8-2* in the *K. phaffii* wild-type CBS7435 on glucose, glycerol, ethanol and methanol relative to the *ACT1* gene. Error bars represent the standard deviations obtained with two biological samples each measured in technical triplicates in two independent experiments. **C** mRNA levels of *CAT8-1* and *CAT8-2* in overexpression mutants and the *K. phaffii* wild-type on ethanol and methanol. Gene expression levels were normalized to the reference gene *ACT1* and quantified relative to wild type (WT) levels (set to 1.0) for each carbon source. Error bars represent the standard deviations of two biological samples each measured in technical triplicates in two independent experiments

Transcript levels of *CAT8-1* in *K. phaffii* wild-type were two- to fourfold increased on ethanol and methanol compared to glucose and glycerol (Fig. 3A) and transcript levels of *CAT8-2* in *K. phaffii* wild-type were higher on glycerol, ethanol, and methanol than on glucose, with more than 500-fold induction on the two alcohols (Fig. 3A). Generally, the expression of *CAT8-1* remained quite low: around 4 and 6% compared to the actin gene (*ACT1)* expression on ethanol and methanol, respectively (Fig. 3B). Expression of *CAT8-2* was much higher: *CAT8-2* reached around 60% of *ACT1* expression on ethanol and methanol (Fig. 3B), which is more than 10-times higher than the expression levels reached by *CAT8-1*.

Regarding the *CAT8* overexpression strains, there was around 15-fold up-regulation of *CAT8-1* in the *CAT8-1* overexpression strain on ethanol, whereas up-regulation was 150-fold in the same strain on methanol compared to the wild-type (Fig. 3C). For *CAT8-2*, we observed 2.34-fold up-regulation in the *CAT8-2* overexpression strain on ethanol and a 40-fold up-regulation on methanol (Fig. 3C). It can therefore be concluded that the overexpression of *CAT8-1* and *CAT8-2* with P_*THI11*_ is stronger on methanol than ethanol. No effect of *CAT8-1* overexpression on *CAT8-2* mRNA levels or vice versa were observed.

### Cat8-1 and Cat8-2 are involved in regulating the expression of genes important for ethanol assimilation

To identify potential target genes of Cat8-1 and Cat8-2, mRNA levels of 13 genes were analyzed in the overexpression and knock-out mutants of *CAT8-1* and *CAT8-2* as well as in the wild-type induced on different carbon sources. The investigated genes were selected because they were either important for the growth of yeasts on gluconeogenic carbon sources and reported to be under the control of Cat8 and/or Sip4 in *S. cerevisiae* and *K. lactis* (*ACS1*, *ADH2*, *ALD4*, *CRC1, FBP1, ICL1, MLS1, PCK1*, and *YAT2*) or involved *K. phaffii* methanol metabolism (*AOX1*, *DAS1*, *PEX5, MXR1*).

**Fig. 4:**
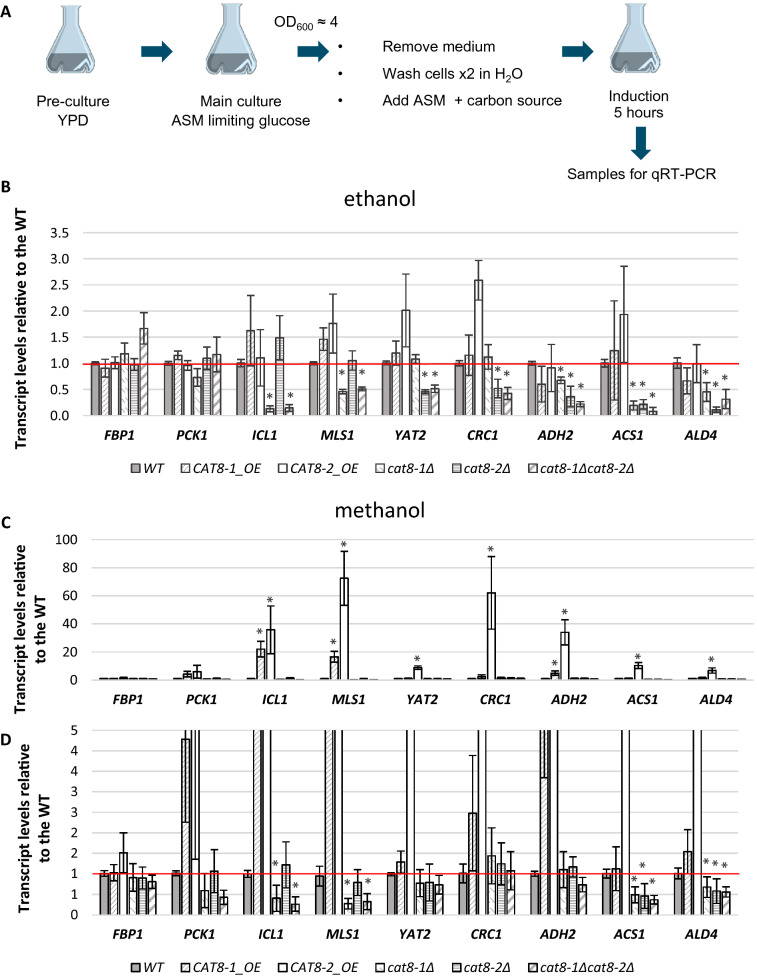
Influence of *CAT8-1* and *CAT8-2* overexpression and knock-out on transcript levels of selected genes in *K. phaffii* induced on ethanol and methanol. **A** Scheme of the sampling for the transcript level analysis. Transcript levels of selected genes in *CAT8-1*_OE, *CAT8-2*_OE, *cat8-1Δ*, *cat8-2Δ* and the double knock-out *cat8-1Δcat8-2Δ* induced on 2% ethanol (**B**) or on 1% methanol (**C**, **D**), determined by qRT-PCR. Gene expression levels were normalized to the reference gene *ACT1* and quantified relative to wild-type (WT) levels (WT set to 1.0, red line). **D** Shows a zoom into **C**. Error bars represent the standard deviations of two independent biological samples each measured in technical triplicates in two independent experiments. The statistically significant differences compared to the WT are indicated with asterisks (Student’s *t* test; ◊*p* < 0.01, *p < 0.001)

The samples for the transcript level analysis were obtained by growing the mutant strains and the wild-type in liquid cultures on minimal medium with limiting glucose to avoid glucose repression in the pre-culture and then shifting the cultures to 2% glucose, 2% glycerol, 1% methanol or 2% ethanol for 5 h to have an induction on either glucose, glycerol, methanol, and ethanol, respectively. Cells were then harvested and RNA was extracted for qRT-PCR (Fig. 4A).

The promoters of the genes selected for the transcript level analysis were screened in silico by MatInspector (Genomatix, Germany) for the presence of the CSRE motif representing the binding site of Cat8 and Sip4. All of the promoters of the selected genes were shown to possess at least one predicted CSRE motif, most of them having more than two of these motifs. In particular, the *CRC1* and the *AOX1* promoter possesses one CSRE, while the *ICL1* and *PEX5* promoters have two CSREs, the *MLS1*, *MXR1,* and *ALD4* promoter three, the *YAT2* and *DAS1* promoters four, the *ADH2* and *FBP1* promoters five and finally the *PCK1* promoter eight predicted CSRE sites.

On glucose and glycerol, no significant difference in transcript levels of the selected genes was observed in the different Cat8 mutant strains compared to the wild-type (data not shown).

On ethanol, the expression of the two gluconeogenic genes *FBP1* and *PCK1* (PP7435_Chr3-0309 and PP7435_Chr1-1542 encoding fructose-1,6-bisphosphatase and phosphoenolpyruvate carboxykinase, respectively) were similar to that of the wild-type in the different mutant strains, showing that Cat8-1 and Cat8-2 are not involved in the regulation of these two genes (Fig. 4B). In *cat8-1Δ*, *cat8-2Δ*, and in the *cat8-1Δcat8-2Δ* double knock-out mutant, the transcript levels of three genes involved in ethanol assimilation, *ADH2* (PP7435_Chr2-0821, encoding alcohol dehydrogenase II), *ACS1* (PP7435_Chr2-0505, encoding acetyl-coenzyme A synthetase I) and *ALD4* (PP7435_Chr2-0787, encoding aldehyde dehydrogenase IV) were all decreased (Fig. 4B), showing that both Cat8-1 and Cat8-2 are involved in the regulation of the expression of these three genes. The expression of two genes from the glyoxylate shunt, *ICL1* (PP7435_Chr1-1123, encoding isocitrate lyase) and *MLS1* (PP7435_Chr4-0820, encoding malate synthase), was decreased in *cat8-1Δ* and in the *cat8-1Δcat8-2Δ* double knock-out mutant but not in *cat8-2Δ* when compared to the wild-type*.* Therefore, only Cat8-1 seems to be necessary for the activation of these genes from the glyoxylate shunt in *K. phaffii*. For two genes from the carnitine shuttle, *YAT2* (PP7435_Chr3-0432, encoding cytosolic carnitine acetyl transferase) and *CRC1* (PP7435_Chr2-0377, encoding carnitine carrier I), the expression was decreased in *cat8-2Δ* and in the *cat8-1Δcat8-2Δ* double knock-out mutant but not in *cat8-1Δ* when compared to the wild-type*.* Therefore, Cat8-2 seems to be necessary for the activation of these genes from the carnitine shuttle. Additionally, the expression of three genes important for methanol assimilation, *AOX1* (PP7435_Chr4-0130, encoding alcohol oxidase I), *DAS1* (PP7435_Chr3-0352, encoding dihydroxyacetone synthase I) and *PEX5* (PP7435_Chr2-0195, encoding a peroxisomal membrane receptor) was assessed in the TF mutant strains on ethanol, but no specific regulation pattern was observed (Supplementary figure S2): due to the very weak expression of these genes on ethanol, the small differences observed in expression were not considered significant. Additionally the expression levels of *MXR1* (PP7435_Chr4-0490) were investigated. *MXR1* expression on ethanol was approximately 60% of the expression on methanol, and was found to be approximately two-fold higher in all *cat8* deletion strains on ethanol (Supplementary figure S2).

Similar to what we observed on ethanol, on methanol we also found the necessity of Cat8-1 for the activation of *ICL1* and *MLS1*, the two genes of the glyoxylate shunt, and the dependence of *ACS1* and *ALD4* on both TFs (Fig. 4C, D). No impact of the knock-outs on the other analyzed genes was observed after induction by methanol. In addition, we observed an increase in transcript levels for all the analyzed genes in the *CAT8-2* overexpression mutant, but except for *ACS1* and *ALD4* there was no significant difference in expression seen in *cat8-2Δ* (Fig. 4C). *ICL1* and *MLS1* expression is also increased in the *CAT8-1* overexpression strain, albeit to a lower level than reached by *CAT8-2* overexpression (Fig. 4C). Also on methanol, there was no impact of any of the Cat8 mutants on the methanol utilization genes (Supplementary figure S2).

### The *CAT8-2* gene expression is autoregulated by the Cat8-2 protein

**Fig. 5:**
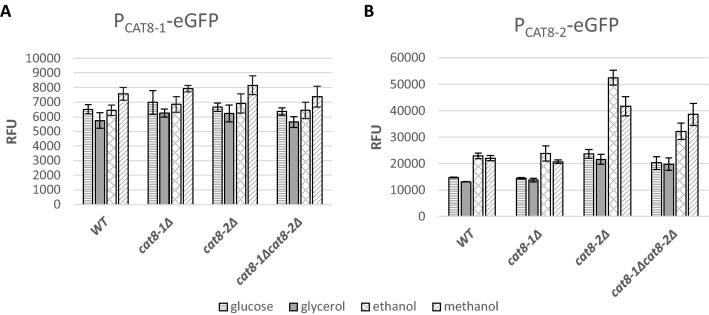
Influence of *CAT8-1* and *CAT8-2* deletions on *CAT8-1* and *CAT8-2* promoter activity. Relative fluorescence units (RFU) obtained for **A** P_*CAT8-1*_-GFP and **B** P_*CAT8-2*_-GFP (1000 bps upstream of the respective *CAT8* gene fused to eGFP as reporter gene) in wild-type CBS7435 and the knock-out mutants *cat8-1Δ*, *cat8-2Δ* and *cat8-1Δcat8-2Δ*. All strains carrying the eGFP reporter construct as well as a non-transformed negative control were grown in biological triplicates on minimal media with limiting glucose and then shifted to glucose, glycerol, ethanol or methanol for 5 h. The eGFP fluorescence was measured by flow cytometry. Mean values and standard deviation for the three biological replicates are presented

In *K. lactis* and *S. cerevisiae*, the Cat8 protein regulates the expression of Sip4, and Sip4 autoregulates the activity of its own promoter (Mehlgarten et al. 2015). In both species, Cat8 transcription factor binding sites (TFBS) are predicted in the *SIP4* promoter regions (Supplementary Figure S3). In order to analyze a possible regulation of the *CAT8-1* and *CAT8-2* genes by the Cat8-1 and Cat8-2 proteins in *K. phaffii*, eGFP reporter strains were generated. The eGFP gene was expressed under the control of the *CAT8-1* and *CAT8-2* promoters, respectively. These constructs were transformed into the different knock-out mutants (*cat8-1Δ*, *cat8-2Δ* and *cat8-1Δcat8-2Δ*) and the wild-type. The obtained strains were cultivated on a minimal medium with limiting glucose and then shifted to either 2% glucose, 2% glycerol, 1% methanol or 2% ethanol for induction. After 5 h induction, the eGFP fluorescence was measured by flow cytometry (Fig. 5).

In all strains and on all the carbon sources, the eGFP fluorescence was lower when the eGFP gene was under the control of the *CAT8-1* promoter P_*CAT8-1*_ compared to when the eGFP gene was under the control of the *CAT8-2* promoter P_*CAT8-2*_ (compare axes in Fig. 5A, B), in accordance with the differences in transcript levels measured by qRT-PCR (Fig. 3B). With the *CAT8-1* promoter, no significant difference was observed in the fluorescence levels of the different strains on the different carbon sources (Fig. 5A). Thus, the expression of *CAT8-1* does not seem to be affected by neither Cat8-1 nor Cat8-2. With the *CAT8-2* promoter in the wild-type, the eGFP fluorescence was lower on glucose and glycerol than on ethanol and methanol (Fig. 5B), which fits to the results obtained for the transcript level analysis of the *CAT8-2* gene on these four carbon sources (Fig. 3A). In the *cat8-1Δ* mutant, the eGFP levels under control of P_*CAT8-2*_ were similar to the ones measured in the wild-type on each of the four carbon sources (Fig. 5B). However, in the *cat8-2Δ* and *cat8-1Δcat8-2Δ* mutants, the eGFP levels under control of P_*CAT8-2*_ were higher than the ones of the wild-type on glucose, glycerol, ethanol, and methanol (Fig. 5B). Therefore, it seems that Cat8-1 is not regulating the expression of *CAT8-2*, but that the Cat8-2 protein is repressing/autoregulating its own promoter on all tested carbon sources.

### The Mig1-2 transcription factor is involved in regulating *CAT8-2* expression

**Fig. 6:**
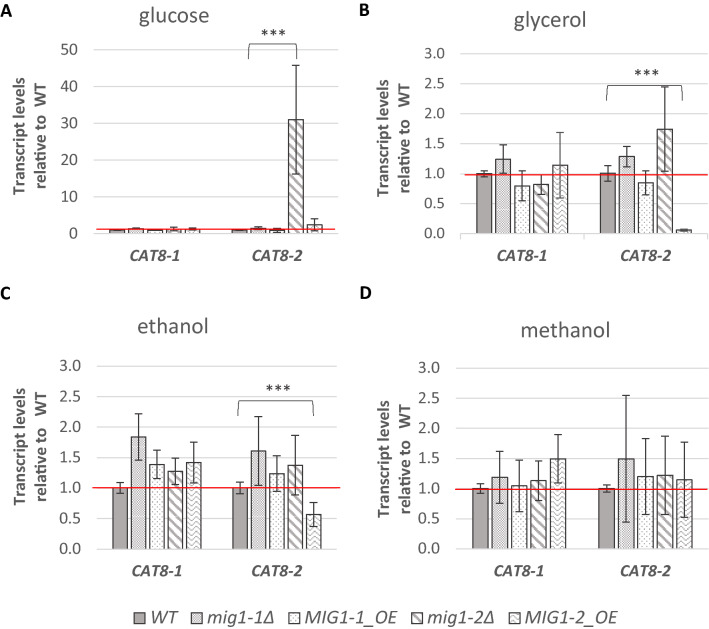
Influence of *MIG1-1* and *MIG1-2* deletion and overexpression on *CAT8-1* and *CAT8-2* transcript levels. Transcript levels of *CAT8-1* and *CAT8-2* in *mig1-1Δ*, *MIG1-1_ OE*, *mig1-2Δ* and *MIG1-2_OE* mutants induced on **A** glucose, **B** glycerol, **C** ethanol or **D** methanol relative to wild-type strain were determined by qRT-PCR. Gene expression levels were normalized to the reference gene *ACT1* and quantified relative to wild-type levels (WT set to 1.0, red line) for each carbon source. Error bars represent the standard deviations of with two independent biological samples each measured in technical triplicates in three independent experiments. The statistically significant differences compared to the WT are indicated with asterisks (Student’s *t* test; ***p < 0.001)

The transcription of *CAT8* was described to be regulated by the transcription factor Mig1 in *S. cerevisiae* (Carlson 1999; Schüller 2003). Correspondingly, there are 4 predicted Mig1 transcription factor binding sites upstream of *S. cerevisiae CAT8* (Supplementary Figure S3). *K. phaffii* possesses two gene homologs of this transcription factor, termed *MIG1-1* (PP7435_Chr4-0661) and *MIG1-2* (PP7435_Chr1-1325). To investigate whether Mig1-1 and Mig1-2 are involved in the transcriptional regulation of *CAT8-1* and *CAT8-2* in *K. phaffii*, their transcript levels were analysed in knock-out and overexpression strains of the two Mig transcription factors (Ata et al. 2017). The Mig1-1 and Mig1-2 mutant strains were first cultivated on minimal medium with limiting glucose until OD_600_ reached 4, and then transferred to 2% glucose, 2% glycerol, 1% methanol or 2% ethanol for induction. After 5 h induction, samples were processed and the transcript levels of *CAT8-1* and *CAT8-2* were measured in the different strains induced on the different carbon sources (Fig. 6).

The *CAT8-1* transcript levels were similar to that of the wild-type in all mutant strains and on all carbon sources (Fig. 6). Therefore *CAT8-1* does not seem to be regulated by neither Mig1-1 nor Mig1-2 in the conditions studied. The expression of *CAT8-2* on the other hand is around 30-fold higher in the *mig1-2Δ* mutant than in the wild-type on glucose (Fig. 6A) and clearly down-regulated on glycerol in the *MIG1-2_OE* mutant (Fig. 6B). Indeed, there are 5 predicted Mig1-TFBS upstream of *CAT8-2*, but only one very distant Mig1-TFBS in the 1000 bps upstream of *CAT8-1* (Supplementary figure S3). Finally, the expression of *CAT8-2* is also lower than in the wild-type on ethanol in the *MIG1-2_OE* mutant (Fig. 6C) and not affected at all on methanol (Fig. 6D). *CAT8-2*, therefore, seems to be repressed in the presence of Mig1-2 on glucose and glycerol, but not on ethanol unless *MIG1-2* is artificially overexpressed. Transcript levels of genes regulated by Cat8-1 and Cat8-2 were also measured in the Mig1-1 and Mig1-2 mutant strains, but no significant difference was observed compared to the wild-type (Supplementary figure S4).

### Investigation of Cat8-1 and Cat8-2 post-translational activation

Cat8 and Sip4 are both activated through phosphorylation in *S. cerevisiae*: Cat8 was shown to be phosphorylated by the yeast homolog of AMPK, the Snf1 kinase complex (Charbon et al. 2004; Randez-Gil et al. 1997), whereas Sip4 was shown to be phosphorylated by both the Snf1 kinase complex (Lesage et al. 1996; Vincent and Carlson 1999) and the Ssn3 kinase (Vincent et al. 2001). Thus, to assess the necessity of post-translational activation of Cat8-1 and/or Cat8-2 in *K. phaffii*, three kinases were selected for deletion: Snf1, Snf1-2 (both homologs of *Sc*Snf1, encoded by PP7435_Chr2-0772 and PP7435_Chr1-0450, respectively), and Ssn3 (homolog of *Sc*Ssn3 encoded by PP7435_Chr1-1091). The generation of the *SNF1-2* and *SSN3* knock-out mutants was successful, but unfortunately, no transformants deleted for *SNF1* could be obtained.

The growth of the *snf1-2Δ* and *ssn3Δ* deletion strains, as well as the *K. phaffii* wild-type, was assessed in liquid cultures with YNB containing either 2% glucose, 2% glycerol, 1% ethanol or 1% methanol (Supplementary figure S5). No difference was observed between the specific growth rates of the *snf1-2Δ* knock-out mutant and the ones of the wild-type on the four-carbon sources tested. However, the specific growth rate of the *ssn3Δ* deletion strain is lower than that of the wild-type on glucose (0.26 h^−1^ for *ssn3Δ* and 0.31 h^−1^ for the wild-type), but not on glycerol, ethanol nor methanol (Supplementary figure S5). These results suggest that the two kinases Snf1-2 and Ssn3 are not necessary for activation of Cat8-1 and Cat8-2 on ethanol.

## Discussion

In this work, we studied the functions of two transcription factors named Cat8-1 and Cat8-2 in *K. phaffii,* which were identified as homologs of the transcription factor Cat8 from *S. cerevisiae* based on sequence similarity (Valli et al. 2016). These two TFs showed differential expression on methanol (Prielhofer et al. 2015) but were otherwise uncharacterized.

### *K. phaffii* Cat8-2 is a Sip4-like protein

**Fig. 7:**
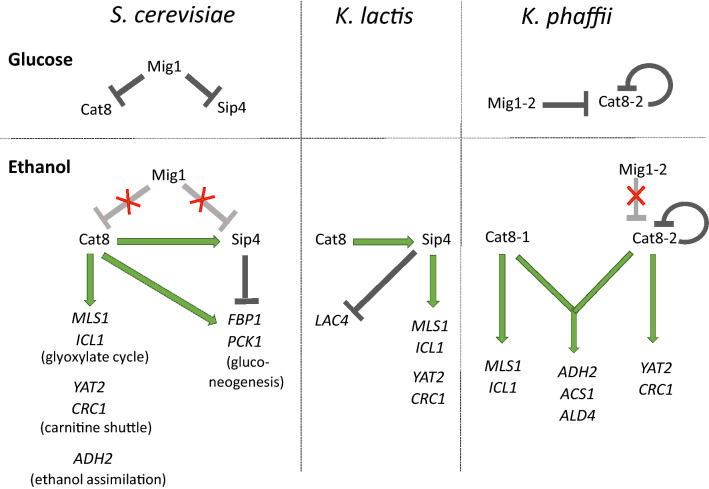
Comparison of the Cat8-Sip4 regulatory networks in *S. cerevisiae*, *K. lactis* and *K. phaffii*. Schematic representation of the regulation by the transcription factors Cat8 and Sip4 for selected genes. Arrows in green indicate transcriptional activation, whereas lines in dark grey indicate transcriptional repression. Elevated repression or derepression is indicated by light grey lines with red crosses

The Cat8 and Sip4 homologous protein sequences used in the phylogenetic analysis showed a conserved DNA binding- and Leucine-zipper dimerization domain, but have a high variation for the rest of the sequences. Based on the inferred phylogenetic tree, the Cat8-1 protein sequence from *K. phaffii* was confirmed to be a Cat8 homolog, whereas Cat8-2 is more closely related to Sip4 protein homologs. While *S. cerevisiae SIP4* was not able to complement the loss of *K. phaffii* Cat8-2, we identified some common features (see Figs. 4, 7) shared by Cat8-2 and Sip4 in the regulation of non-fermentable carbon sources in *K. phaffii*, *S. cerevisiae* and *K. lactis*. Taken all our findings together, Cat8-2 can be considered a Sip4-like protein in *K. phaffii*.

### Role of Cat8-1 and Cat8-2 in the regulation of ethanol utilization

When shifted to a gluconeogenic carbon source, yeast cells undergo a massive reprogramming of their gene expression, including genes involved in gluconeogenesis, the glyoxylate cycle, and tricarboxylic acid cycle. Ethanol is metabolized by an alcohol dehydrogenase (encoded by *ADH2*) to acetaldehyde, which is subsequently converted into acetate by aldehyde dehydrogenase (*ALD*). Acetate is then transformed into acetyl-Coenzyme A by acetyl-CoA synthetase (*ACS*) in the cytoplasm. Then, acetyl-CoA must be transferred to the mitochondria for the production of energy (Schmalix and Bandlow 1993; Stemple et al. 1998). Two pathways exist to transport acetyl-CoA into the mitochondria: (1) acetyl-CoA is converted into glyoxylate cycle intermediates which are transported to the mitochondria (Palmieri et al. 1997) and (2) acetyl-CoA is converted into acetylcarnitine, which is transported into mitochondria via the carnitine shuttle. In addition to the carnitine shuttle and the glyoxylate cycle, gluconeogenesis is fundamental for the growth of non-fermentable carbon sources: it is essential for the production of glucose-6-phosphate, which is crucial for cell growth (Barnett and Entian 2005).

Both Cat8-1 and Cat8-2 were shown to be essential for the growth of *K. phaffii* on ethanol (Fig. 2), specifically, both transcription factors are required for the activation of *ADH2*, *ALD4* and *ACS1*, three genes encoding enzymes important for assimilation of ethanol (Fig. 4A). But while Cat8-1 seems to be necessary for the activation of the glyoxylate cycle (by regulating *MLS1* and *ICL1*), Cat8-2 seems to be necessary for the activation of the carnitine shuttle (by regulating *YAT2* and *CRC1*). In contrast, the gluconeogenic genes *FBP1* and *PCK1* are not regulated by neither Cat8-1 nor Cat8-2 (Fig. 4). Furthermore, we could show that the *CAT8-2* gene is clearly repressed in the presence of the Mig1-2 transcription factor (a homolog of *S. cerevisiae* Mig1) on glucose and to a lesser extent also on glycerol (Fig. 6), and repressed/autoregulated by the Cat8-2 protein on glucose, glycerol, ethanol, and methanol (Fig. 5).

Altogether, we can observe three major differences in the regulation of genes for ethanol utilization by Cat8/Cat8-1 and Sip4/Cat8-2 in the three yeast species *S. cerevisiae*, *K. lactis* and *K. phaffii* (Fig. 7): (1) Cat8 is involved in the regulation of the gluconeogenesis in *S. cerevisiae* but neither in *K. lactis* nor in *K. phaffii*, (2) the expression of Sip4 is activated by Cat8 in *S. cerevisiae* and *K. lactis* but not in *K. phaffii*, and (3) the dissimilar importance of Cat8 and Sip4 in the different yeasts: although the same set of genes are targeted by Cat8/Sip4 and Cat8-1/Cat8-2 (except for the gluconeogenesis genes), the specific TF regulating the individual genes varies in the different species.

### Regulation of the gluconeogenesis

In *S. cerevisiae*, Cat8 and Sip4 are involved in the regulation of genes of the gluconeogenesis, therefore knocking out the genes encoding these two TFs results in a growth defect on glycerol (Hedges et al. 1995; Rahner et al. 1996). In *K. phaffii* as well as in *K. lactis*, when *CAT8-1* and *CAT8-2* (or *CAT8* and *SIP4*) are knocked-out, there is no impairment of the growth on glycerol. In addition, transcript level analysis in *K. phaffii* showed that *CAT8-1* and *CAT8-2* deletions did not have a major influence on *FBP1* and *PCK1* expression on any of the tested carbon sources (Fig. 4 for ethanol and methanol, not shown for glucose and glycerol). Therefore, the regulation of gluconeogenesis seems to be achieved by a different regulatory network in *K. phaffii* compared to *S. cerevisiae*. One TF that could potentially play an important role in such a regulatory network is Rds2, which was shown to have partially overlapping functions with Cat8 in *S. cerevisiae* and to be a major regulator of gluconeogenesis. Specifically, Rds2 was shown to directly activate the expression of the gluconeogenic genes while repressing the negative regulators of this pathway, possibly through binding of the CSREs (Soontorngun et al. 2007). To our knowledge, the homolog of Rds2 in *K. phaffii* (PP7435_Chr2-1080) was not studied so far but could be involved in the regulation of gluconeogenesis in this yeast.

#### *Activation of* SIP4 *expression by CAT8*

In both *S. cerevisiae* and *K. lactis*, Cat8 has an important role in activating Sip4 expression on ethanol. Despite the fact that both *CAT8-1* and *CAT8-2* promoters carry three and two CSRE motifs, respectively (Supplementary File 2), this phenomenon is not observed in *K. phaffii*, suggesting that rewiring of the regulatory network of the carbon metabolism has happened somewhere during evolution.

##### Direct targets of CAT8 and SIP4 vary in the different yeast species

In *S. cerevisiae*, Cat8 is the main activator for the growth of gluconeogenic carbon sources. In fact, the *SIP4* deletion mutants do not exhibit any growth defect on any carbon source tested so far (Lesage et al. 1996), and the role of *Sc*Sip4 is still unclear. *Sc*Cat8, on the other hand, was shown to activate the expression of genes from gluconeogenesis, the glyoxylate cycle, the ethanol assimilation pathway, and the carnitine shuttle. In *K. lactis*, Cat8 activates the expression of Sip4 but does not seem to directly activate the expression of genes important for the growth of gluconeogenic carbon sources. Instead, *Kl*Sip4 is important for the regulation of the glyoxylate cycle and the carnitine shuttle. In *K. phaffii*, some genes seem to be specifically regulated only in the presence of either Cat8-1 or Cat8-2, while others seem to be overlapping targets of these two TFs. Indeed, Cat8-1 seems to be necessary for the activation of the genes from the glyoxylate cycle on ethanol, acetate, and methanol, while Cat8-2 seems to be important for the activation of genes from the carnitine shuttle on ethanol. As only *cat8-1Δ*, but not *cat8-2Δ,* showed clearly reduced growth on acetate, it seems that on acetate the glyoxylate cycle is the major active pathway for acetyl-CoA shuttling. In addition, Cat8-1 and Cat8-2 are both involved in the regulation of *ADH2*, *ALD4* and *ACS1*, three genes important for the conversion of ethanol to acetyl-CoA (Fig. 4), although the contribution of each TF for the regulation of these three genes remains to be investigated.

### Cat8-1 and Cat8-2 possibly recognize different subsets of CSRE motifs

It was shown in *S. cerevisiae* that mutant CSREs show differential activation by Cat8 and Sip4 (Roth et al. 2004). In addition, the purified DNA binding domain of the TF Rds2 was shown to bind to the CSREs of the *PCK1* and *FBP1* promoters (Soontorngun et al. 2007). It was hypothesized that these three TFs bind subsets of CSREs with diverging affinities, which would allow for the regulation of both distinct and common target genes. This phenomenon could also be present in *K. phaffii*: transcript-level analysis showed that on methanol in the *CAT8-2* overexpression strain, there are increased transcript levels of *YAT2* and *CRC1* as well as *ICL1* and *MLS1*, two genes mainly regulated by Cat8-1, whereas in the *CAT8-1* overexpression, there is an increase only in *ICL1* and *MLS1* transcript levels (Fig. 4). This suggests that Cat8-1 and Cat8-2 could each have specific binding sites, but that, when Cat8-2 is present in high amounts, like on methanol and when strongly overexpressed, Cat8-2 could also bind a motif otherwise specific for Cat8-1. However, further experiments such as chromatin immunoprecipitation (ChiP) assay need to be performed in the future to investigate the specific DNA binding sites of Cat8-1 and Cat8-2.

In *S. cerevisiae*, *ADH2* (Walther and Schüller 2001) and *ACS1* (Kratzer and Schüller 1997) were reported to be synergistically activated by Cat8 and Adr1. The sole homolog of Adr1 in *K. phaffii* is termed Mxr1 (methanol expression regulator 1) due to its function as activator of methanol utilization genes such as *AOX1* (Lin-Cereghino et al. 2006; Kranthi et al. 2010). While Mxr1 is essential for the utilization of methanol, mutants lacking Mxr1 are still able to grow on other carbon sources including ethanol, albeit with a prolonged generation time (Lin-Cereghino et al. 2006). Mxr1 has recently also been implicated with the regulation of *ACS1* of *K. phaffii* grown on acetate in complex YP-containing media (Sahu and Rangarajan 2016). Expression levels of *MXR1* are approximately 40% lower on ethanol than on methanol, and there is some indication that Cat8-1 and Cat8-2 are involved in repressing *MXR1* expression on ethanol (Supplementary Figure S2), however, we do not know if this is due to direct binding of the TFs to the CSRE present in the *MXR1* promoter region or not. Additionally, there seems to be some regulatory effect of the overexpression of one transcription factor on the expression of the other on methanol, but the increased *MXR1* levels were not reflected by higher levels of Mxr1 target genes such as *AOX1*, *DAS1* or *PEX5* (Supplementary Figure S2). This fits the recent observation that integration of at least one *Sc*Cat8-TFBS into the *AOX1* promoter was needed in order to make the promoter responsive to ethanol (Ergün et al. 2020). Vice versa, no effect of Mxr1 overexpression on P_*ADH2*_-GFP (Ergün et al. 2019) was seen on ethanol. Future research should be directed towards the interrelation of Cat8-1 and Cat8-2 with the Adr1-homolog Mxr1 and other carbon-source responsive transcriptional regulators in *K. phaffii*.

### Mechanisms of activation of Cat8 family members

In the presence of glucose, expression of *CAT8* and *SIP4* is inhibited by the TF Mig1 and the Ssn6/Tup1 corepressor complex in *S. cerevisiae* (Carlson 1999; Schüller 2003). *K. phaffii* possesses two homologs of *Sc*Mig1: Mig1-1 and Mig1-2. These two TFs were previously shown to be involved in the regulation of the methanol metabolism in *K. phaffii*: derepression of *AOX1* was observed in *mig1-1Δ* and *mig1-1Δmig1-2Δ* knock-out strains on glycerol (Wang et al. 2017), and the genes of the methanol metabolism, as well as the peroxisomes biogenesis pathway, were upregulated on glycerol in *mig1-1Δmig1-2Δ* (Shi et al. 2018). Finally, Mig1-1 and Mig1-2 localized in the nucleus of cells grown on glucose or glycerol, but when *K. phaffii* cells were transferred to methanol, Mig1-1 and Mig1-2 predominantly localized to the cytoplasm (Wang et al. 2017).

In our experiments, we observed repression of *CAT8-2* on glucose and glycerol by Mig1-2, but not on the two alcohols. No activation of the Cat8-2 target genes was observed in the Mig1-1 and Mig1-2 deletion strains, which can be explained by the involvement of further TFs in the regulation of these genes on glucose and/or glycerol.

We furthermore wanted to assess the necessity of post-translational activation of Cat8-1 and/or Cat8-2, since Cat8 and Sip4 are both activated upon phosphorylation by the Snf1 and/or the Ssn3 kinases in *S. cerevisiae* (Charbon et al. 2004; Randez-Gil et al. 1997; Lesage et al. 1996; Vincent and Carlson, 1999; Vincent et al. 2001). The homolog of *Sc*Ssn3 and the two putative homologs of *Sc*Snf1 present in *K. phaffii*, Snf1, and Snf1-2, were therefore selected for deletion. Unfortunately, the generation of the *SNF1* knock-out mutant was not successful. Since disruption of Snf1 was also not possible in the approach by Shen et al. (Shen et al. 2016), we think Snf1 to be essential in *K. phaffii* under the investigated conditions. Additionally, Snf1 was already reported to be essential in other non-*Saccharomyces* yeast species (Hedbacker and Carlson 2008).

No difference was seen in the growth of the *snf1-2Δ* and *ssn3Δ* deletion strains compared to the wild-type on ethanol (Supplementary figure S4), hence these two kinases do not seem to play a major role in the regulation of ethanol utilization in *K. phaffii*, and are also probably not involved in the activation of Cat8-1 and Cat8-2 on this carbon source. Further investigations regarding the post-translational modifications of Cat8-1 and Cat8-2 would be necessary in order to elucidate if they are indeed activated via phosphorylation in *K. phaffii*, as observed for *S. cerevisiae*.

## Conclusions

The present study elucidated the requirement of the two Cat8 homologs Cat8-1 and Cat8-2 for activation of the ethanol assimilation pathway in *K. phaffii*, and their differential involvement in regulating the carnitine shuttle and the glyoxylate shunt, respectively. Phylogenetic analysis places Cat8-2 among the Sip4-like proteins. However, different to *S. cerevisiae* and *K. lactis*, *CAT8-2* expression is not regulated by the Cat8-1 TF, but autoregulated by its own gene product. Furthermore, *CAT8-2* is repressed in the presence of Mig1-2 on glucose. Despite their transcriptional induction on both ethanol and methanol, no direct impact on methanol utilization genes could be observed in the Cat8 mutant strains. Interestingly, however, the CSRE motif, which was shown to be recognized by Cat8 and Sip4 in *S. cerevisiae* and *K. lactis,* was predicted by MatInspector to be present in around 90% of the total promoters in *K. phaffii* (Supplementary material 3). Contrary to what is described for *cat8Δ* and *sip4Δ* in *S. cerevisiae*, knocking-out *CAT8-1* and *CAT8-2* also influences the ability of *K. phaffii* to tolerate osmotic stress and cell wall damaging agents (Supplementary figure S6). These results suggest that these two TFs might have additional targets and a broader role in *K. phaffii,* both of which remain to be investigated.

## Supplementary Information

Below is the link to the electronic supplementary material.

Supplementary file1 (PDF 1172 KB)

Supplementary file2List of all K. phaffii CBS7435 genes with CSREs predicted by Matinspector 1000 bps upstream of their start codon (sheet Matinspector) and enrichment of gene ontology (GO) “biological functions” within theses genes (sheet GO_analysis) (XLSX 222 KB)

## Data Availability

All data generated or analyzed during this study are included in this published article and its supplementary information files.

## References

[CR1] Ata O, Prielhofer R, Gasser B, Mattanovich D, Calik P (2017). Transcriptional engineering of the glyceraldehyde-3-phosphate dehydrogenase promoter for improved heterologous protein production in *Pichia pastoris*. Biotechnol Bioeng.

[CR2] Barnett JA, Entian KD (2005). A history of research on yeasts 9: regulation of sugar metabolism. Yeast.

[CR3] Capella-Gutierrez S, Silla-Martinez JM, Gabaldon T (2009). trimAl: a tool for automated alignment trimming in large-scale phylogenetic analyses. Bioinformatics.

[CR4] Carlson M (1999). Glucose repression in yeast. Curr Opin Microbiol.

[CR5] Charbon G, Breunig KD, Wattiez R, Vandenhaute J, Noël-Georis I (2004). Key role of Ser562/661 in Snf1-dependent regulation of Cat8p in *Saccharomyces cerevisiae* and *Kluyveromyces lactis*. Mol Cell Biol.

[CR6] De Vit MJ, Waddle JA, Johnston M (1997). Regulated nuclear translocation of the Mig1 glucose repressor. Mol Biol Cell.

[CR7] Delic M, Mattanovich D, Gasser B (2013). Repressible promoters—a novel tool to generate conditional mutants in *Pichia pastoris*. Microb Cell Fact.

[CR8] Engler C, Kandzia R, Marillonnet S (2008). A one pot, one step, precision cloning method with high throughput capability. PLoS ONE.

[CR9] Ergün BG, Gasser B, Mattanovich D, Çalık P (2019). Engineering of alcohol dehydrogenase 2 hybrid-promoter architectures in *Pichia pastoris* to enhance recombinant protein expression on ethanol. Biotechnol Bioeng.

[CR10] Ergün BG, Demir İ, Özdamar TH, Gasser B, Mattanovich D, Çalık P (2020). Engineered deregulation of expression in yeast with designed hybrid-promoter architectures in coordination with discovered master regulator transcription factor. Adv Biosyst.

[CR11] Foley G, Sützl L, D'Cunha SA, Gillam EMJ, Bodén M (2019). SeqScrub: a web tool for automatic cleaning and annotation of FASTA file headers for bioinformatic applications. Biotechniques.

[CR12] Gasser B, Prielhofer R, Marx H, Maurer M, Nocon J, Steiger M, Puxbaum V, Sauer M, Mattanovich D (2013). *Pichia pastoris*: protein production host and model organism for biomedical research. Future Microbiol.

[CR13] Gassler T, Heistinger L, Mattanovich D, Gasser B, Prielhofer R (2019). CRISPR/Cas9-mediated homology directed genome editing in *Pichia pastoris*. Methods Mol Biol.

[CR14] Georis I, Krijger JJ, Breunig KD, Vandenhaute J (2000). Differences in regulation of yeast gluconeogenesis revealed by Cat8p-independent activation of *PCK1* and *FBP1* genes in *Kluyveromyces lactis*. Mol Gen Genet.

[CR15] Guindon S, Dufayard JF, Lefort V, Anisimova M, Hordijk W, Gascuel O (2010). New algorithms and methods to estimate maximum-likelihood phylogenies: assessing the performance of PhyML 3.0. Syst Biol.

[CR16] Hardie DG, Carling D, Carlson M (1998). The AMP-activated/SNF1 protein kinase subfamily: metabolic sensors of the eukaryotic cell?. Annu Rev Biochem.

[CR17] Hartner FS, Glieder A (2006). Regulation of methanol utilisation pathway genes in yeasts. Microb Cell Fact.

[CR18] Haurie V, Perrot M, Mini T, Jenö P, Sagliocco F, Boucherie H (2001). The transcriptional activator Cat8p provides a major contribution to the reprogramming of carbon metabolism during the diauxic shift in *Saccharomyces cerevisiae*. J Biol Chem.

[CR19] Hedbacker K, Carlson M (2008). SNF1/AMPK pathways in yeast. Front Biosci.

[CR20] Hedges D, Proft M, Entian KD (1995). *CAT8*, a new zinc cluster-encoding gene necessary for derepression of gluconeogenic enzymes in the yeast *Saccharomyces cerevisiae*. Mol Cell Biol.

[CR21] Hiesinger M, Roth S, Meissner E, Schuller HJ (2001). Contribution of Cat8 and Sip4 to the transcriptional activation of yeast gluconeogenic genes by carbon source-responsive elements. Curr Genet.

[CR22] Katoh K, Standley DM (2013). MAFFT Multiple Sequence Alignment Software Version 7: improvements in performance and usability. Mol Biol Evol.

[CR23] Kranthi BV, Kumar HR, Rangarajan PN (2010). Identification of Mxr1p-binding sites in the promoters of genes encoding dihydroxyacetone synthase and peroxin 8 of the methylotrophic yeast *Pichia pastoris*. Yeast.

[CR24] Kratzer S, Schüller HJ (1997). Transcriptional control of the yeast acetyl-CoA synthetase gene, *ACS1*, by the positive regulators *CAT8* and *ADR1* and the pleiotropic repressor *UME6*. Mol Microbiol.

[CR25] Landes N, Gasser B, Vorauer-Uhl K, Lhota G, Mattanovich D, Maurer M (2016). The vitamin-sensitive promoter P_*THI11*_ enables pre-defined autonomous induction of recombinant protein production in *Pichia pastoris*. Biotechnol Bioeng.

[CR26] Lesage P, Yang X, Carlson M (1996). Yeast SNF1 protein kinase interacts with SIP4, a C6 zinc cluster transcriptional activator: a new role for SNF1 in the glucose response. Mol Cell Biol.

[CR27] Lin-Cereghino GP, Godfrey L, de la Cruz BJ, Johnson S, Khuongsathiene S, Tolstorukov I, Yan M, Lin-Cereghino J, Veenhuis M, Subramani S, Cregg JM (2006). Mxr1p, a key regulator of the methanol utilization pathway and peroxisomal genes in *Pichia pastoris*. Mol Cell Biol.

[CR28] Mehlgarten C, Krijger JJ, Lemnian I, Gohr A, Kasper L, Diesing AK, Grosse I, Breunig KD (2015). Divergent evolution of the transcriptional network controlled by Snf1-Interacting protein Sip4 in budding yeasts. PLoS ONE.

[CR29] Monteiro PT, Oliveira J, Pais P, Antunes M, Palma M, Cavalheiro M, Galocha M, Godinho CP, Martins LC, Bourbon N, Mota MN, Ribeiro RA, Viana R, Sá-Correia I, Teixeira MC (2020). YEASTRACT+: a portal for cross-species comparative genomics of transcription regulation in yeasts. Nucleic Acids Res.

[CR30] Palmieri L, Lasorsa FM, De Palma A, Palmieri F, Runswick MJ, Walker JE (1997). Identification of the yeast *ACR1* gene product as a succinate-fumarate transporter essential for growth on ethanol or acetate. FEBS Lett.

[CR31] Prielhofer R, Barrero JJ, Steuer S, Gassler T, Zahrl R, Baumann K, Sauer M, Mattanovich D, Gasser B, Marx H (2017). GoldenPiCS: a Golden Gate-derived modular cloning system for applied synthetic biology in the yeast *Pichia pastoris*. BMC Syst Biol.

[CR32] Prielhofer R, Cartwright SP, Graf AB, Valli M, Bill RM, Mattanovich D, Gasser B (2015). *Pichia pastoris* regulates its gene-specific response to different carbon sources at the transcriptional, rather than the translational, level. BMC Genomics.

[CR33] Qi K, Zhong JJ, Xia XX (2014). Triggering respirofermentative metabolism in the crabtree-negative yeast *Pichia guilliermondii* by disrupting the *CAT8* gene. Appl Environ Microbiol.

[CR34] Rahner A, Schöler A, Martens E, Gollwitzer B, Schüller HJ (1996). Dual influence of the yeast Cat1p (Snf1p) protein kinase on carbon source-dependent transcriptional activation of gluconeogenic genes by the regulatory gene *CAT8*. Nucleic Acids Res.

[CR35] Ramirez MA, Lorenz MC (2009). The transcription factor homolog *CTF1* regulates {beta}-oxidation in *Candida albicans*. Eukaryot Cell.

[CR36] Randez-Gil F, Bojunga N, Proft M, Entian KD (1997). Glucose derepression of gluconeogenic enzymes in *Saccharomyces cerevisiae* correlates with phosphorylation of the gene activator Cat8p. Mol Cell Biol.

[CR37] Rodicio R, López ML, Cuadrado S, Cid AF, Redruello B, Moreno F, Heinisch JJ, Hegewald AK, Breunig KD (2008). Differential control of isocitrate lyase gene transcription by non-fermentable carbon sources in the milk yeast *Kluyveromyces lactis*. FEBS Lett.

[CR38] Roth S, Kumme J, Schüller HJ (2004). Transcriptional activators Cat8 and Sip4 discriminate between sequence variants of the carbon source-responsive promoter element in the yeast *Saccharomyces cerevisiae*. Curr Genet.

[CR39] Ruchala J, Kurylenko OO, Soontorngun N, Dmytruk KV, Sibirny AA (2017). Transcriptional activator Cat8 is involved in regulation of xylose alcoholic fermentation in the thermotolerant yeast *Ogataea* (*Hansenula*) *polymorpha*. Microb Cell Fact.

[CR40] Sahu U, Rangarajan PN (2016). Regulation of acetate metabolism and acetyl Co-A synthetase 1 (*ACS1*) expression by methanol expression regulator 1 (Mxr1p) in the methylotrophic yeast *Pichia pastoris*. J Biol Chem.

[CR41] Schmalix W, Bandlow W (1993). The ethanol-inducible *YAT1* gene from yeast encodes a presumptive mitochondrial outer carnitine acetyltransferase. J Biol Chem.

[CR42] Schüller HJ (2003). Transcriptional control of nonfermentative metabolism in the yeast *Saccharomyces cerevisiae*. Curr Genet.

[CR43] Shen W, Kong C, Xue Y, Liu Y, Cai M, Zhang Y, Jiang T, Zhou X, Zhou M (2016). Kinase screening in *Pichia pastoris* identified promising targets involved in cell growth and alcohol oxidase 1 promoter (P_*AOX1*_) regulation. PLoS ONE.

[CR44] Shi L, Wang X, Wang J, Zhang P, Qi F, Cai M, Zhang Y, Zhou X (2018). Transcriptome analysis of Δ*mig1*Δ*mig2* mutant reveals their roles in methanol catabolism, peroxisome biogenesis and autophagy in methylotrophic yeast *Pichia pastoris*. Genes Genomics.

[CR45] Soontorngun N, Larochelle M, Drouin S, Robert F, Turcotte B (2007). Regulation of gluconeogenesis in *Saccharomyces cerevisiae* is mediated by activator and repressor functions of Rds2. Mol Cell Biol.

[CR46] Stemple CJ, Davis MA, Hynes MJ (1998). The facC gene of *Aspergillus nidulans* encodes an acetate-inducible carnitine acetyltransferase. J Bacteriol.

[CR47] Tachibana C, Yoo JY, Tagne JB, Kacherovsky N, Lee TI, Young ET (2005). Combined global localization analysis and transcriptome data identify genes that are directly coregulated by Adr1 and Cat8. Mol Cell Biol.

[CR48] Todd RB, Andrianopoulos A (1997). Evolution of a fungal regulatory gene family: the Zn(II)2Cys6 binuclear cluster DNA binding motif. Fungal Genet Biol.

[CR49] Todd RB, Kelly JM, Davis MA, Hynes MJ (1997). Molecular characterization of mutants of the acetate regulatory gene facB of *Aspergillus nidulans*. Fungal Genet Biol.

[CR50] Todd RB, Andrianopoulos A, Davis MA, Hynes MJ (1998). FacB, the *Aspergillus nidulans* activator of acetate utilization genes, binds dissimilar DNA sequences. EMBO J.

[CR51] Toussaint M, Conconi A (2006). High-throughput and sensitive assay to measure yeast cell growth: a bench protocol for testing genotoxic agents. Nat Protoc.

[CR52] Turcotte B, Liang XB, Robert F, Soontorngun N (2010). Transcriptional regulation of nonfermentable carbon utilization in budding yeast. FEMS Yeast Res.

[CR53] Valli M, Tatto NE, Peymann A, Gruber C, Landes N, Ekker H, Thallinger GG, Mattanovich D, Gasser B, Graf AB (2016). Curation of the genome annotation of *Pichia pastoris* (*Komagataella phaffii*) CBS7435 from gene level to protein function. FEMS Yeast Res.

[CR54] Vincent O, Carlson M (1998). Sip4, a Snf1 kinase-dependent transcriptional activator, binds to the carbon source-responsive element of gluconeogenic genes. EMBO J.

[CR55] Vincent O, Carlson M (1999). Gal83 mediates the interaction of the Snf1 kinase complex with the transcription activator Sip4. EMBO J.

[CR56] Vincent O, Kuchin S, Hong SP, Townley R, Vyas VK, Carlson M (2001). Interaction of the Srb10 kinase with Sip4, a transcriptional activator of gluconeogenic genes in *Saccharomyces cerevisiae*. Mol Cell Biol.

[CR57] Walther K, Schüller HJ (2001). Adr1 and Cat8 synergistically activate the glucose-regulated alcohol dehydrogenase gene *ADH2* of the yeast *Saccharomyces cerevisiae*. Microbiol.

[CR58] Wang J, Wang X, Shi L, Qi F, Zhang P, Zhang Y, Zhou X, Song Z, Cai M (2017). Methanol-independent protein expression by *AOX1* promoter with trans-acting elements engineering and glucose-glycerol-shift induction in *Pichia pastoris*. Sci Rep.

