# Supplementary material for: Two homologs of the Cat8 transcription factor are involved in the regulation of ethanol utilization in Komagataella phaffii
Source: Curr Genet. 2021 Mar 16;67(4):641–61. doi: 10.1007/s00294-021-01165-4 (PMC8254726; doi:10.1007/s00294-021-01165-4)
Supplement: Supplementary file 1 — Supplementary file1 (PDF 1172 KB) [file 294_2021_1165_MOESM1_ESM.pdf]

Supplementary Material to

Two homologs of the Cat8 transcription factor are involved in regulating ethanol utilization in *Komagataella phaffii*

Diane Barbay[1,2], Monika Mačáková[2], Leander Sützl[3], Sonakshi De[1,2], Diethard Mattanovich[1,2], Brigitte Gasser[1,2*]

1. Austrian Centre of Industrial Biotechnology (ACIB), Vienna, Austria
2. Department of Biotechnology, University of Natural Resources and Life Sciences (BOKU), Vienna, Austria
3. Department of Food Technology, University of Natural Resources and Life Sciences (BOKU), Vienna, Austria

Supplementary figure S1: Growth of the *cat8-1Δ::CAT8-1-HA* and *cat8-2Δ::CAT8-2-HA* on ethanol. Plasmids carrying the promoter regions (upstream 1000 bp regions), C-terminally tagged coding sequences and terminator regions of *CAT8-1* or *CAT8-2* were transformed into *K. phaffii cat8-1Δ* (for Cat8-1-HA) and *cat8-2Δ* (for Cat8-2-HA) deletion strains. The growth of the obtained strains was assessed on 1% ethanol.

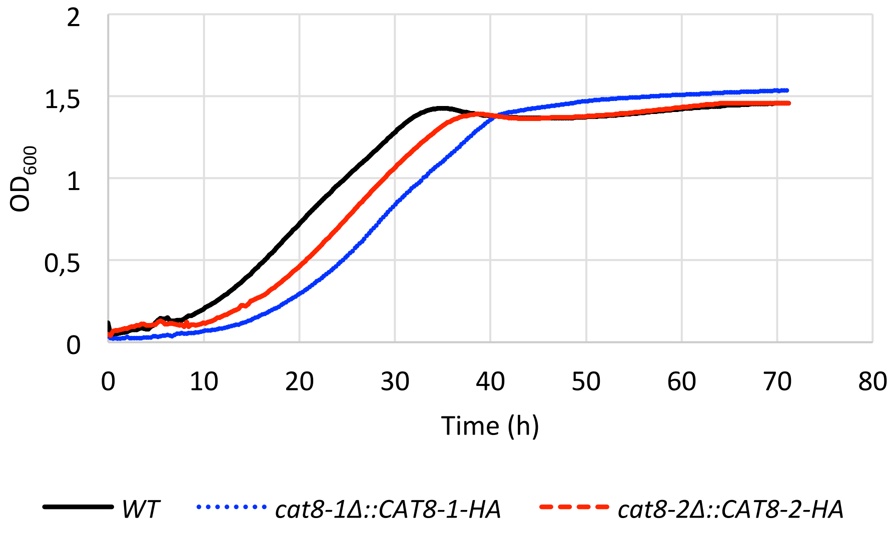

Supplementary figure S2: Influence of *CAT8-1* and *CAT8-2* overexpression and knock-out on transcript levels of methanol utilization genes in *K. phaffii* induced on ethanol or methanol. Transcript levels of *AOX1*, *DAS1, PEX5* and *MXR1* in *CAT8-1_OE*, *CAT8-2_OE*, *cat8-1Δ*, *cat8-2Δ* and *cat8-1Δcat8-2Δ* induced on 2% ethanol and 1% methanol, determined by qRT-PCR. Gene expression levels were normalized to the reference gene *ACT1* and quantified relative to wild type levels (WT, set to 1.0). Error bars represent the standard deviations of two independent biological samples each measured in technical triplicates in up to two independent experiments.

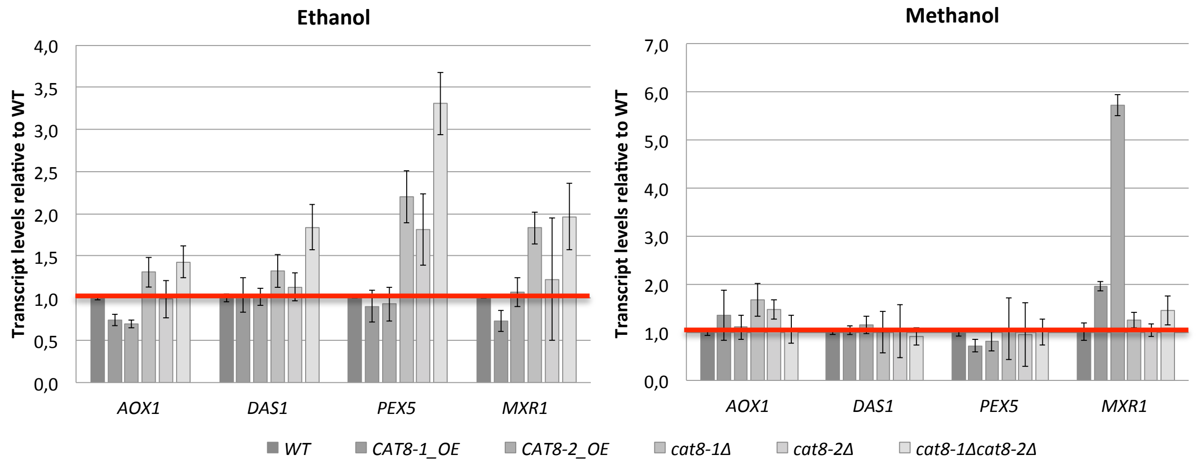

Supplementary figure S3: Prediction of transcription factor binding sites (TFBS) in CAT8/CAT8-1 and SIP4/CAT8-2 promoter regions. TFBS were predicted by yeastract-plus.org in the regions 1000 bps upstream of the respective genes in *S. cerevisiae*, *K. lactis* and *K. phaffii*. TFBS for Mig1/2, Cat8/Sip4 (CSRE) and Adr1/Mxr1 are depicted; the full list of predicted TFBS is given as supplementary material 2.

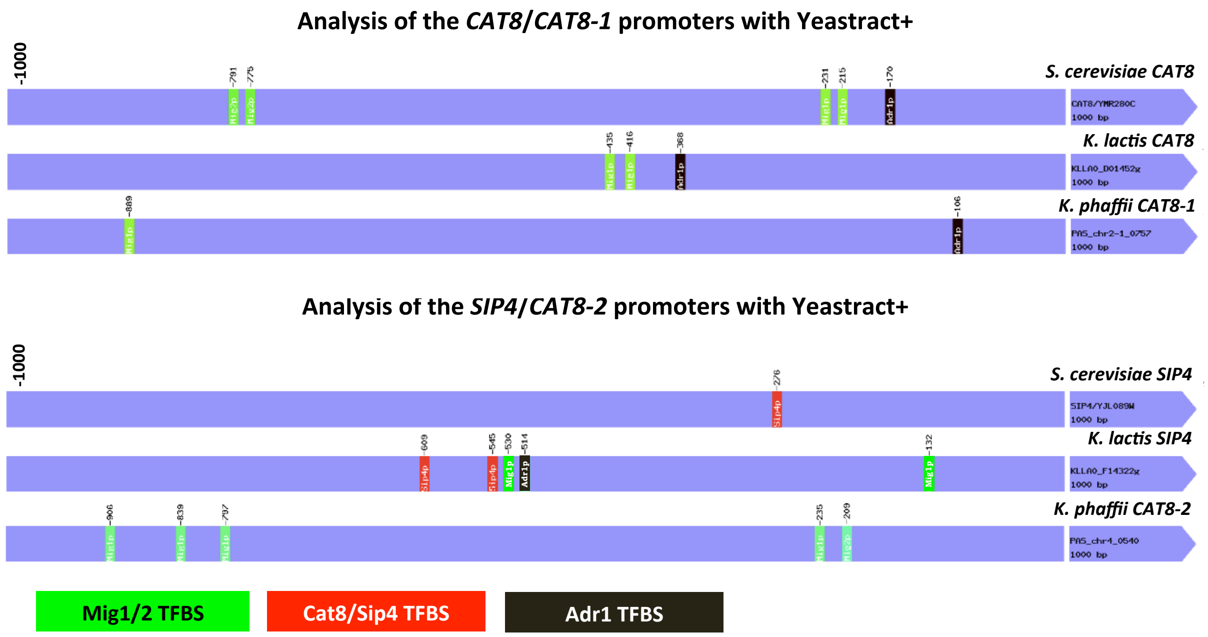

CAT8 / *CAT8-1* promoter				
**Species**	**TF**	**Consensus**	**Position**	**Strand**
K. phaffii	Adr1p	TTGGRG	-106	R
	Mig1p	SYGGRG	-889	R
S. cerevisiae	Adr1p	TTGGRG	-170	R
	Mig1p Mig2p	CCCCRC	-791	R
	Mig1p Mig2p	CCCCRC	-775	R
	Mig1p	SYGGRG	-231	F
	Mig1p	SYGGRG	-215	F
K. lactis	Adr1p	TTGGRG	-368	F
	Mig1p	SYGGRG	-435	F
	Mig1p	SYGGRG	-416	R

SIP4 / *CAT8-2* promoter				
**Species**	**TF**	**Consensus**	**Position**	**Strand**
K. phaffii	Mig1p Mig2p	CCCCRC	209	R
	Mig1p	SYGGRG	-906	F
	Mig1p	SYGGRG	-839	F
	Mig1p	SYGGRG	-797	F
	Mig1p	SYGGRG	-235	F
S. cerevisiae	Cat8p Sip4p	NCCDTYNVN	-276	F
K. lactis	Adr1p	TTGGRG	514	R
	Cat8p Sip4p	NCCDTYNVN	-609	F
	Cat8p Sip4p	NCCDTYNVN	545	R
	Cat8p Sip4p	YCCNYTNRR	545	R
	Mig1p	SYGGRG	-132	F
	Mig1p	SYGGRG	530	R

Supplementary figure S4: Influence of *MIG1-1* and *MIG1-2* deletion and overexpression on the transcript levels of selected genes. Transcript levels of *ADH2*, *ICL1*, *YAT2* and *AOX1* in *mig1-1Δ*, *MIG1-1_ OE*, *mig1-2Δ* and *MIG1-2_OE* mutants induced on (A) glucose, (B) glycerol, (C) ethanol or (D) methanol relative to wild-type strain were determined by qRT-PCR. Gene expression levels were normalized to the reference gene *ACT1* and quantified relative to wild type levels (WT, set to 1.0) for each carbon source. Mean values and standard deviation for two independent biological samples each measured in technical triplicates in three independent experiments are presented.

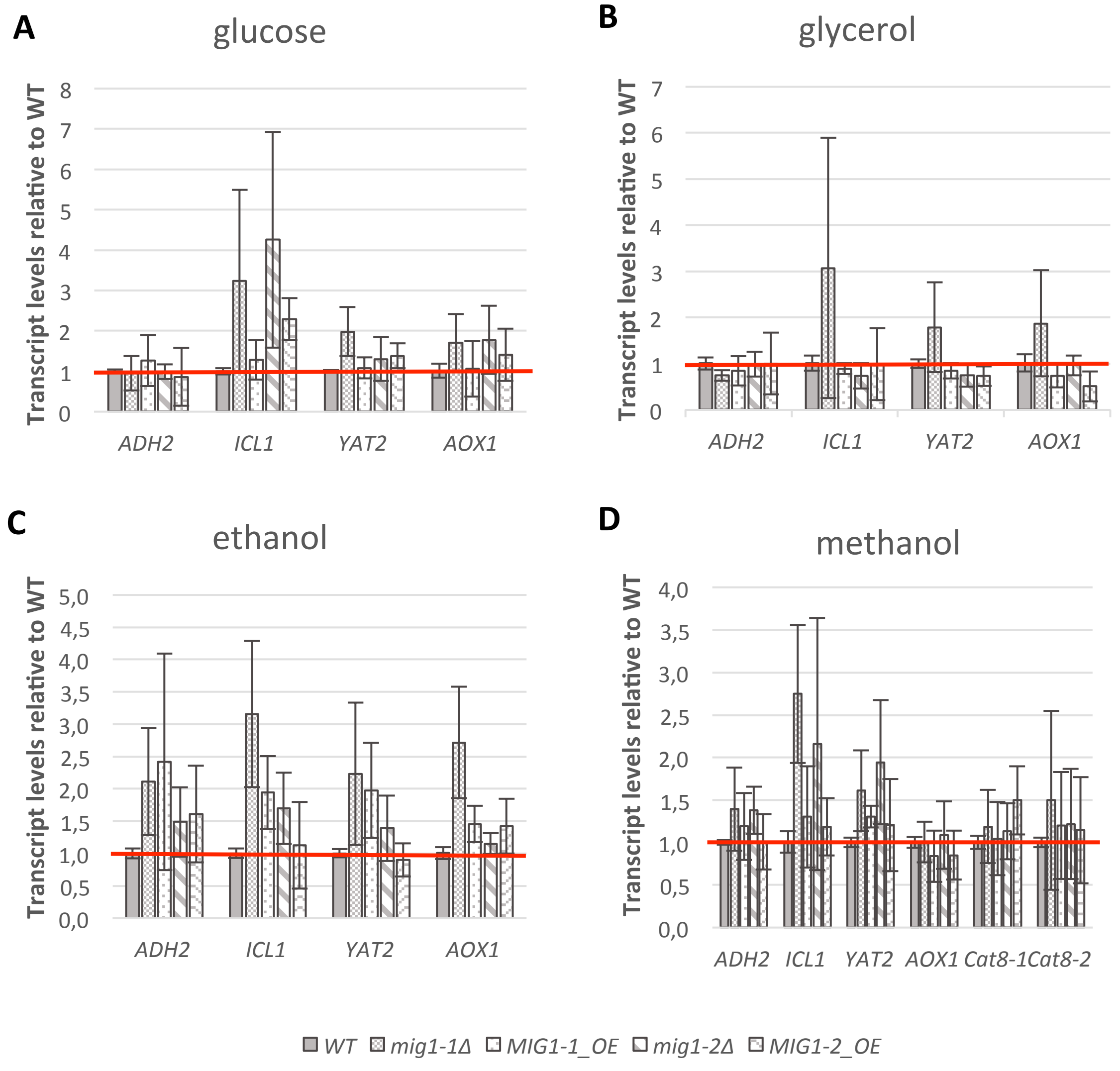

Supplementary figure S5: Influence of *SNF1-2* and *SSN3* deletion on carbon source utilization. Growth rates of the *snf1-2Δ* and *ssn3Δ* knock-out mutants and the *K. phaffii* wild-type (WT) on YNB without thiamine with 2% glucose, 2% glycerol, 1% methanol and 1% ethanol. Error bars represent the standard deviations of three independent biological samples each measured in technical triplicates. Statistically significant differences compared to the WT are indicated with asterisks (Student's t-test; * $p<0.05$, ** $p<0.01$, *** $p<0.001$).

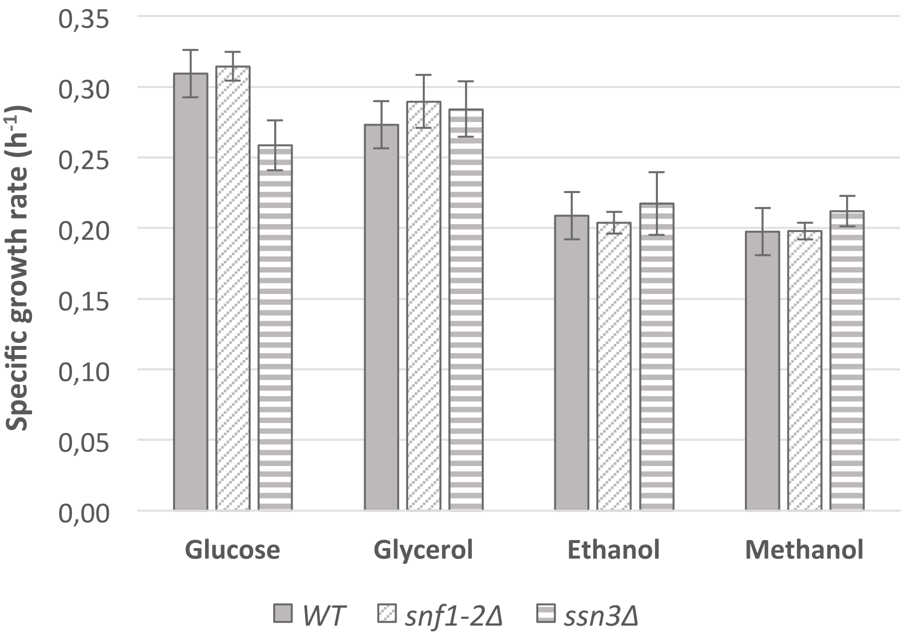

Supplementary figure S6: Spotting assay of *cat8-1Δ* and *cat8-2Δ* on different stressors. On each plate the *K. phaffii* wild-type (WT) and 4 individual clones of the Cat8 mutants were spotted in serial dilutions. The control plate is YNB-agar supplemented with 2% glucose. It was incubated in the same conditions than the plate testing the growth in the presence of a stressor. Only stressors leading to effects on growth are shown.

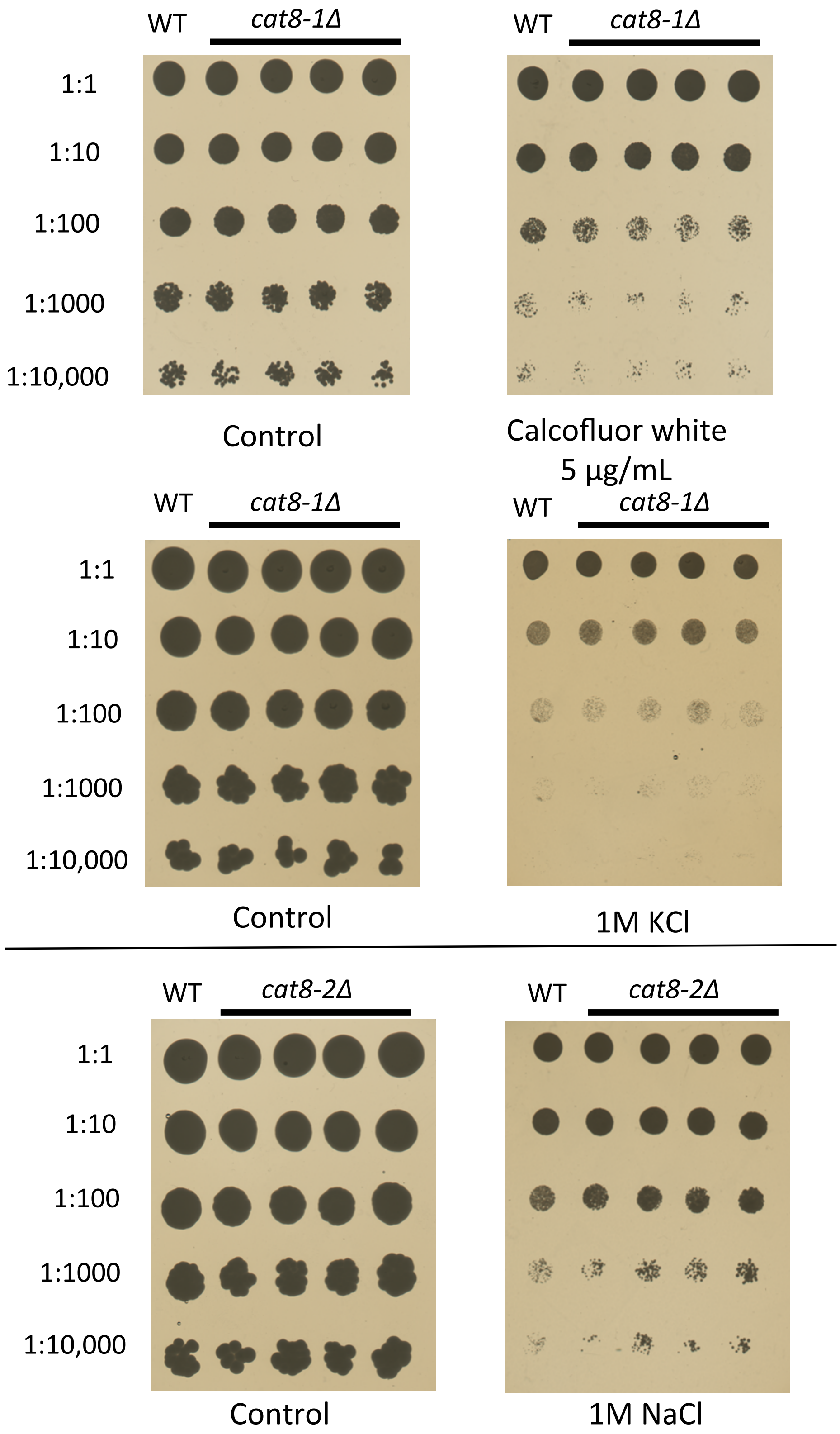

Supplementary table 1: Primers used for the generation of the overexpression strains.

Name	Target	Sequence	Purpose
**CAT8-1_Primer_OE_Fw**	*CAT8-1*	GATTAGGTCTCCCATGATGCCGGAGGAAC	GG cloning
**CAT8-1_Primer_OE_Rv**	*CAT8-1*	GATTAGGTCTCCAAGCCTAAAGTCCGAATAAACTCCC	GG cloning
**CAT8-2_OE_gBlock**	*CAT8-2*	GATTAGGTCTCCTACAAAAGACCATCTTCAATAGTCAAACAAGAACCAACAATCAACCCGAGGTCAAATGGCACTAACACCGATAGCAATCTATTCGATACCTTTAATGATTCTATCAAAGGCTCTTTGAATAACGGTTTGAAGAAGTTGAAAGATATCAGATGCAATTCTGTCGTGGAAAGATCTCACTCTTCCCAAAGAAATGATTTCTTGATGGATCAAGAGGACAGTATAACCAAGGAGACAATCAACTTTTCTGAGCTTTTCACCTGCGGAACTCCAACTGCGTCTCAGAGTATTGACAGATCTCCCAAGTCACTGCTGTTAAATGACTTAGCTATAGCTCCCGATACTTTGGTCATCAAACCAGACGCTGAAGATCTGGACAGATTGAAAAACAAAATCAGATCTGTCAAATCAACTGTTCACTAGGCTTCGAGACCGATTA	GG cloning
**CAT8-2_OE_Fw1**	*CAT8-2*	GATTAGGTCTCGCATGAAAGAGAACCAAGCCTCC	GG cloning
**CAT8-2_OE_Rv1**	*CAT8-2*	GAGTCTAGACTATTGTTATCTTCTGCAAATTTTTCGTTAAG	GG cloning
**CAT8-2_OE_Fw2**	*CAT8-2*	CTTAACGAAAAATTTGCAGAAGATAACAATAGTCTAGACTC	GG cloning
**CAT8-2_OE_Rv2**	*CAT8-2*	CTTGGGTCAATGTGTAGACGGAGTGAAG	GG cloning
**CAT8-2_OE_Fw3**	*CAT8-2*	CTTCACTCCGTCTACACATTGACCCAAG	GG cloning
**CAT8-2_OE_Rv3**	*CAT8-2*	GAACATATTCATCAAATCTTCATGAGCAGCAGAG	GG cloning
**CAT8-2_OE_Fw4**	*CAT8-2*	CTCTGCTGCTCATGAAGATTTGATGAATATGTTC	GG cloning
**CAT8-2_OE_Rv4**	*CAT8-2*	GATTAGGTCTCTTGTAACTTCCAAAAAGCGAG	GG cloning

Supplementary table 2: Primers used for the generation of the knock-out strains.

Name	Target	Sequence	Purpose
**CAT8-2_KO_HR1_Fw**	CAT8-2_HR1	GATTAGGTCTCCCATGCGTCTCGGGCAGAAAGAAAGTACTAATATG	GG cloning
**CAT8-2_KO_HR1_Rv**	CAT8-2_HR1	GATTAGGTCTCCTAAACAAAATAAGAGATACTCTAAAAAAACAAG	GG cloning
**CAT8-2_KO_HR2_Fw1**	CAT8-2_HR2	GATTAGGTCTCCTTTAGAGTAAAAGAGTTCCCAAATGAATG	GG cloning
**CAT8-2_KO_HR2_Rv1**	CAT8-2_HR2	TAATCTACGGTCTAGTGATGGTGATC	GG cloning
**CAT8-2_KO_HR2_Fw2**	CAT8-2_HR2	ATCACCATCACTAGACCGTAGATTAC	GG cloning
**CAT8-2_KO_HR2_Rv2**	CAT8-2_HR2	GATTAGGTCTCGAAGCCGTCTCAGGATGGTCCAAGCGTC	GG cloning
**CAT8-1_KO_HR1_Fw**	CAT8-1_HR1	GATTAGGTCTCCCATGGAAGACCTGCTCGTCGTGTCCTAAC	GG cloning
**CAT8-1_KO_HR1_Rv**	CAT8-1_HR1	GATTAGGTCTCCGGGAGAGCTAGTGAGAAAC	GG cloning
**CAT8-1_KO_HR2_Fw1**	CAT8-1_HR2	GATTAGGTCTCCTCCCGAGAAGGTAGAGCGGATAG	GG cloning
**CAT8-1_KO_HR2_Rv1**	CAT8-1_HR2	CAATGAATGGACTCTCTTAACGGTTAATC	GG cloning
**CAT8-1_KO_HR2_Fw2**	CAT8-1_HR2	GATTAACCGTTAAGAGAGTCCATTCATTG	GG cloning

**CAT8-1_KO_HR2_Rv2**	CAT8-1_HR2	GATTAGGTCTCCAAGCGAAGACCAGAAAGATGCTGAGTGAC	GG cloning
**CAT8-2_gRNA_1**	CAT8-2	GATAGGTCTCCCATGCTTTGTCTGATGAGTCCGTGAGGACGAAACGAGTAAGCTCGTC	gRNA assembly
**CAT8-2_gRNA_2**	CAT8-2	AAACGAGTAAGCTCGTCACAAAGAAGTAACAAATAAGttttagagctagaaatagcaag	gRNA assembly
**CAT8-1_gRNA_1**	CAT8-1	GATAGGTCTCCCATGTACTAACTGATGAGTCCGTGAGGACGAAACGAGTAAGCTCGTC	gRNA assembly
**CAT8-1_gRNA_2**	CAT8-1	AAACGAGTAAGCTCGTCTTAGTACGAAGCCAGCTCGCgttttagagctagaaatagcaag	gRNA assembly
**CAT8-2_KO_outHR_Fw**	CAT8-2_OutHR	CGGGAGAACACTTTTGATG	Colony PCR KO
**CAT8-2_KO_outHR_Rv**	CAT8-2_OutHR	CTGTTGGGTGTCTATCTAGG	Colony PCR KO
**CAT8-1_KO_OutHR_Fw**	CAT8-1_OutHR	AATACCAATAACCAGCACC	Colony PCR KO
**CAT8-1_KO_OutHR_Rv**	CAT8-1_OutHR	ATTATCGCCTTTTTCCTGAG	Colony PCR KO
**Snf1-2_HA1_Fw**	SNF1-2	GATTAGGTCTCCCATGCGTCTCAAAATCTCCGACTCTAGACT	GG cloning
**Snf1-2_HA1_Rv**	SNF1-2	GATTAGGTCTCCGAAGGAGCGAGTTATACGA	GG cloning
**Snf1-2_HA2_Fw**	SNF1-2	GATTAGGTCTCACTTCTAAGTTGATTCGTAAATAGCCATCG	GG cloning
**Snf1-2_HA2_Rv**	SNF1-2	GATTAGGTCTCGAAGCCGTCTCGAAAAAGGAAATGCAGCGAG	GG cloning
**Ssn3_Srb10_HA1_Fw**	SSN3	GATTAGGTCTCCCATGCGTCTCCAAACTCTCTCCTCTGATACC	GG cloning
**Ssn3_Srb10_HA1_Rv**	SSN3	GATTAGGTCTCCGTGATTTTATTGTGGCTGGG	GG cloning
**Ssn3_Srb10_HA2_Fw**	SSN3	GATTAGGTCTCATCACTAAAGGTAGCTGAAAGGGTAG	GG cloning
**Ssn3_Srb10_HA2_Rv**	SSN3	GATTAGGTCTCGAAGCCGTCTCTTGTTGTTGCTGGTGGTG	GG cloning
**Snf1-2_gRNA_1**	SNF1-2	GATAGGTCTCCCATGGAGTTGCTGATGAGTCCGTGAGGACGAAACGAGTAAGCTCGTC	gRNA assembly
**Snf1-2_gRNA_2**	SNF1-2	AAACGAGTAAGCTCGTCCAACTCCACGTCTACAGACTgttttagagctagaaatagcaag	gRNA assembly
**Ssn3_gRNA_1**	SSN3	GATAGGTCTCCCATGTGAGGACTGATGAGTCCGTGAGGACGAAACGAGTAAGCTCGTC	gRNA assembly
**Ssn3_gRNA_2**	SSN3	AAACGAGTAAGCTCGTCTCCTCATTGGCTGCTGCGTTgttttagagctagaaatagcaag	gRNA assembly
**Snf1-2_KO_OutHR_Fw**	Snf1-2_KO_OutHR	CGAACATTGACGAGGGAAA	Colony PCR KO
**Snf1-2_KO_OutHR_Rv**	Snf1-2_KO_OutHR	CACCAACAACTCCACCAC	Colony PCR KO
**Ssn3_KO_OutHR_Fw**	Ssn3_KO_OutHR	GCTTCTGCATTGAAATC	Colony PCR KO
**Ssn3_KO_OutHR_Rv**	Ssn3_KO_OutHR	TCCCGTTTTACTCACAC	Colony PCR KO

Supplementary table 3: List of primers used for the gene copy number determination.

Name	Target	Sequence	Purpose
**CAT8-2_qPCR_Fw**	*CAT8-2*	GGTCTTCAGCGTCTGGTT	qRT-PCR
**CAT8-2_qPCR_Rv**	*CAT8-2*	ACTTTTCTGAGCTTTTCACCTG	qRT-PCR
**CAT8-1_qPCR_Fw**	*CAT8-1*	TAATGTATCTCCTCCCAATAGTGAAAG	qRT-PCR
**CAT8-1_qPCR_Rv**	*CAT8-1*	GTCCGAATAAACTCCCAGCAG	qRT-PCR

Supplementary table 4: List of primers used for the transcript level analysis.

Name	Target	Sequence	Purpose
**ACT1_up**	*ACT1*	CCTGAGGCTTTGTTCCACCCATCT	qRT-PCR
**ACT1_low**	*ACT1*	GGAACATAGTAGTACCACCGGACATAACGA	qRT-PCR
**CAT8-2_qRT-PCR_Fw**	*CAT8-2*	GGTCTTCAGCGTCTGGTT	qRT-PCR
**CAT8-2_qRT-PCR_Rv**	*CAT8-2*	ACTTTTCTGAGCTTTTCACCTG	qRT-PCR
**CAT8-1_qRT-PCR_Fw**	*CAT8-1*	TAATGTATCTCCTCCCAATAGTGAAAG	qRT-PCR
**CAT8-1_qRT-PCR_Rv**	*CAT8-1*	GTCCGAATAAACTCCCAGCAG	qRT-PCR
**AOX1_qRT-PCR_Fw**	*AOX1*	TTGAAGGTTGGTGACTTGTCC	qRT-PCR
**AOX1_qRT-PCR_Rv**	*AOX1*	AGGAACAGTCATGTCTAAGGC	qRT-PCR
**DAS1_qRT-PCR_Fw**	*DAS1*	CGGTAAGTCTCTTCCTGTTG	qRT-PCR
**DAS1_qRT-PCR_Rv**	*DAS1*	TTGGTTTTCCCTTCAAGTCG	qRT-PCR
**PEX5_qRT-PCR_Fw**	*PEX5*	CTCTCCTATTCATACCCAAAAATGC	qRT-PCR
**PEX5_qRT-PCR_Rv**	*PEX5*	AGGTTGAAGGTGTTGATGC	qRT-PCR
**ICL1_qRT-PCR_Fw**	*ICL1*	CAGAAATGGTCAGGAGCCG	qRT-PCR
**ICL1_qRT-PCR_Rv**	*ICL1*	AATGGTCCTTGAATTGATCTTCAG	qRT-PCR
**ADH2_qRT-PCR_Fw**	*ADH2*	CCAGCCTCCATCTGTTCGTA	qRT-PCR
**ADH2_qRT-PCR_Rv**	*ADH2*	GTTCTGAAGTCCATCGAGATCAAG	qRT-PCR
**MLS1_qRT-PCR_Fw**	*MLS1*	ACTGGCGAGAAGATCACA	qRT-PCR
**MLS1_qRT-PCR_Rv**	*MLS1*	CTCCACTAATCTCCTTCTTCAAAC	qRT-PCR
**YAT2_qRT-PCR_Fw**	*YAT2*	GGCAGTGCAACCAACTTA	qRT-PCR
**YAT2_qRT-PCR_Rv**	*YAT2*	TGGAAGAAAGAGGTGGAGAGTGAA	qRT-PCR
**CRC1_qRT-PCR_Fw**	*CRC1*	ATGCCTTAACCCCACCTTT	qRT-PCR
**CRC1_qRT-PCR_Rv**	*CRC1*	ACAATGGCAGGAGGTTTT	qRT-PCR
**FBP1_qRT-PCR_Fw**	*FBP1*	TGAAGAAACCCCAAGCAAAC	qRT-PCR
**FBP1_qRT-PCR_Rv**	*FBP1*	TGGAGTCTGCTGGATAGC	qRT-PCR
**PCK1_qRT-PCR_Fw**	*PCK1*	CCAACCTTCGGTCTACAAAT	qRT-PCR
**PCK1_qRT-PCR_Rv**	*PCK1*	CTCGGTGTTGAAGTTGTCT	qRT-PCR
**ACS1_qRT-PCR_Fw**	*ACS1*	GGTTTTGGCTGGAGAGGAAGA	qRT-PCR
**ACS1_qRT-PCR_Rv**	*ACS1*	TTGCGGGCATCCCTTTT	qRT-PCR
**ALD4_qRT-PCR_Fw**	*ALD4*	CGGTCTTGCTGCTGGTAT	qRT-PCR
**ALD4_qRT-PCR_Rv**	*ALD4*	TTTGGTGGAAATCGTTGTAGGT	qRT-PCR
**MIG1-2_qRT-PCR_Fw**	*MIG1-2*	ACACACACAGGGGAAAAAC	qRT-PCR
**MIG1-2_qRT-PCR_Rv**	*MIG1-2*	CTGCCCTTTCTCACAGTTG	qRT-PCR
**MIG1-1_qRT-PCR_Fw**	*MIG1-1*	TCCTCCATCCCATATTGATTCC	qRT-PCR
**MIG1-1_qRT-PCR_Rv**	*MIG1-1*	TGGAAACGGATTTTGGAAAGAG	qRT-PCR

Supplementary table 5: Primers used for cloning the *CAT8-1* and *CAT8-2* promoter regions P_{CAT8-1} and P_{CAT8-2}

Name	Target	Sequence	Purpose
**CAT8-1_GG_prom_Fw**	P_{CAT8-1}	GATTAGAAGACCCGGAGACTCCAACAGAGAGTGAATTG	Prom CAT8-1_eGFP
**CAT8-1_GG_prom_Rv**	P_{CAT8-1}	GATTAGAAGACCCCATGTTTAAGGAGATGAATCGAGACAC	Prom CAT8-1_eGFP
**CAT8-2_GG_prom_Fw**	P_{CAT8-2}	GATTAGAAGACCCGGAGGGTGATCATGAGTTGCATCC	Prom CAT8-2_eGFP
**CAT8-2_GG_prom_Rv**	P_{CAT8-2}	GATTAGAAGACCCCATGGAACTGGGTGTAGTGGGTAAAAC	Prom CAT8-2_eGFP

Supplementary material 1: List of protein identifiers (NCBI accession numbers) of sequences used for the phylogenetic analyses of Cat8-1 and Cat8-2. The order corresponds to the occurrence of the sequences in the phylogenetic tree. All sequences are named according to their taxonomy. Characterized sequences and *K. phaffii* Cat8-1 and Cat8-2 are highlighted in bold.

Cat8-2_Phaffomycetaceae_and_Pichiaceae_clade

XP_011276078.1_Saccharomycetales_Phaffomycetaceae_Wickerhamomyces_Wickerhamomyces_ciferrii
CEP22994.1_Saccharomycetales_Phaffomycetaceae_Cyberlindnera_Cyberlindnera_jadinii
CDR44725.1_Saccharomycetales_Phaffomycetaceae_Cyberlindnera_Cyberlindnera_fabianii
Cat8_2_XP_002493979.1_Saccharomycetales_Phaffomycetaceae_Komagataella_Komagataella_phaffii_GS115
ANZ78000.1_Saccharomycetales_Phaffomycetaceae_Komagataella_Komagataella_pastoris
OWB49142.1_Saccharomycetales_Pichiaceae_Ogataea_Candida_boidinii
OWB81660.1_Saccharomycetales_Pichiaceae_Ogataea_Candida_boidinii
ODV87249.1_Saccharomycetales_Pichiaceae_Ogataea_Candida_arabinofermentans_NRRL_YB-2248
XP_018208536.1_Saccharomycetales_Pichiaceae_Ogataea_Ogataea_polymorpha
XP_013937395.1_Saccharomycetales_Pichiaceae_Ogataea_Ogataea_parapolymorpha_DL-1
GAV28280.1_Saccharomycetales_Pichiaceae_Pichia_Pichia_membranifaciens
XP_019017762.1_Saccharomycetales_Pichiaceae_Pichia_Pichia_membranifaciens_NRRL_Y-2026
KGK38021.1_Saccharomycetales_Pichiaceae_Pichia_Pichia_kudriavzevii
OUT21345.1_Saccharomycetales_Pichiaceae_Pichia_Pichia_kudriavzevii
AWU75108.1_Saccharomycetales_Pichiaceae_Pichia_Pichia_kudriavzevii

Sip4_Saccharomycetaceae_and_Saccharomycodaceae_clade

XP_004180554.1_Saccharomycetales_Saccharomycetaceae_Tetrapisispora_Tetrapisispora_blattae_CBS_6284
SSD61869.1_Saccharomycetales_Saccharomycodaceae_Saccharomycodes_Saccharomycodes_ludwigii
XP_017986333.1_Saccharomycetales_Saccharomycetaceae_Eremothecium_Eremothecium_sinecaudum
XP_003647533.1_Saccharomycetales_Saccharomycetaceae_Eremothecium_Eremothecium_cymbalariae_DBVPG7215
AGO13243.1_Saccharomycetales_Saccharomycetaceae_Saccharomycetaceae_sp._Ashbya_aceri
NP_985643.2_Saccharomycetales_Saccharomycetaceae_Eremothecium_Eremothecium_gossypii_ATCC_10895
XP_022675308.1_Saccharomycetales_Saccharomycetaceae_Kluyveromyces_Kluyveromyces_marxianus_DMKU3-1042
CDO96066.1_Saccharomycetales_Saccharomycetaceae_Kluyveromyces_Kluyveromyces_dobzhanskii_CBS_2104
Sip4_CAE00852.1_Saccharomycetales_Saccharomycetaceae_Kluyveromyces_Kluyveromyces_lactis
XP_455723.1_Saccharomycetales_Saccharomycetaceae_Kluyveromyces_Kluyveromyces_lactis
SCW00936.1_Saccharomycetales_Saccharomycetaceae_Lachancea_Lachancea_fermentati
SCV00608.1_Saccharomycetales_Saccharomycetaceae_Lachancea_Lachancea_mirantina
XP_002552880.1_Saccharomycetales_Saccharomycetaceae_Lachancea_Lachancea_thermotolerans_CBS_6340
CUS20723.1_Saccharomycetales_Saccharomycetaceae_Lachancea_Lachancea_quebecensis
SCU90333.1_Saccharomycetales_Saccharomycetaceae_Lachancea_Lachancea_nothofagi_CBS_11611
SCU83125.1_Saccharomycetales_Saccharomycetaceae_Lachancea_Lachancea_dasiensis_CBS_10888
SCU78707.1_Saccharomycetales_Saccharomycetaceae_Lachancea_Lachancea_meyersii_CBS_8951
XP_022629831.1_Saccharomycetales_Saccharomycetaceae_Lachancea_Lachancea_lanzarotensis
SCU77698.1_Saccharomycetales_Saccharomycetaceae_Lachancea_Lachancea_sp._CBS_6924
XP_001643936.1_Saccharomycetales_Saccharomycetaceae_Vanderwaltozyma_Vanderwaltozyma_polyspora_DSM_70294
XP_003687217.1_Saccharomycetales_Saccharomycetaceae_Tetrapisispora_Tetrapisispora_phaffii_CBS_4417
XP_018221239.1_Saccharomycetales_Saccharomycetaceae_Saccharomyces_Saccharomyces_eubayanus
EJT44735.1_Saccharomycetales_Saccharomycetaceae_Saccharomyces_Saccharomyces_kudriavzevii_IFO_1802
EJS43144.1_Saccharomycetales_Saccharomycetaceae_Saccharomyces_Saccharomyces_arboricola_H-6
AJR71411.1_Saccharomycetales_Saccharomycetaceae_Saccharomyces_Saccharomyces_cerevisiae_YJM1447
KQC43050.1_Saccharomycetales_Saccharomycetaceae_Saccharomyces_Saccharomyces_sp._boulardii
AJR60093.1_Saccharomycetales_Saccharomycetaceae_Saccharomyces_Saccharomyces_cerevisiae_YJM195
Sip4_CAA89382.1_Saccharomycetales_Saccharomycetaceae_Saccharomyces_Saccharomyces_cerevisiae
AJP39617.1_Saccharomycetales_Saccharomycetaceae_Saccharomyces_Saccharomyces_cerevisiae_YJM1078

PTN14399.1_Saccharomycetales_Saccharomycetaceae_Saccharomyces_Saccharomyces_cerevisiae
EHN06472.1_Saccharomycetales_Saccharomycetaceae_Saccharomyces_Saccharomyces_cerevisiae_x_Saccharomyces_kudriavzevii_VIN7
XP_003980212.1_Saccharomycetales_Saccharomycetaceae_Naumovozyma_Naumovozyma_dairenensis_CBS_421
XP_003673880.1_Saccharomycetales_Saccharomycetaceae_Naumovozyma_Naumovozyma_castellii_CBS_4309
XP_022462873.1_Saccharomycetales_Saccharomycetaceae_Kazachstania_Kazachstania_naganishii_CBS_8797
SMN21725.1_Saccharomycetales_Saccharomycetaceae_Kazachstania_Kazachstania_saulgeensis
XP_003954721.1_Saccharomycetales_Saccharomycetaceae_Kazachstania_Kazachstania_africana_CBS_2517
XP_448919.1_Saccharomycetales_Saccharomycetaceae_Nakaseomyces_Candida_glabrata
SLM12920.1_Saccharomycetales_Saccharomycetaceae_Nakaseomyces_Candida_glabrata
GAV56003.1_Saccharomycetales_Saccharomycetaceae_Zygosaccharomyces_Zygosaccharomyces_rouxii
GAV51207.1_Saccharomycetales_Saccharomycetaceae_Zygosaccharomyces_Zygosaccharomyces_rouxii
CDH15222.1_Saccharomycetales_Saccharomycetaceae_Zygosaccharomyces_Zygosaccharomyces_bailii_ISA1307
SJM83910.1_Saccharomycetales_Saccharomycetaceae_Zygosaccharomyces_Zygosaccharomyces_bailii
AQZ12288.1_Saccharomycetales_Saccharomycetaceae_Zygosaccharomyces_Zygosaccharomyces_parabailii
CDF90410.1_Saccharomycetales_Saccharomycetaceae_Zygosaccharomyces_Zygosaccharomyces_bailii_CLIB_213

Cat8_Various-yeasts_and_Aspergillus_clade

Cat8_CBF88979.1_Eurotiales_Aspergillaceae_Aspergillus_Aspergillus_nidulans_FGSC_A4
ODV92727.1_Saccharomycetales_Trigonopsidaceae_Tortispora_Tortispora_caseinolytica_NRRL_Y-17796
ODQ74993.1_Saccharomycetales_Lipomycetaceae_Lipomyces_Lipomyces_starkeyi_NRRL_Y-11557
ODQ66190.1_Saccharomycetales_Nadsonia_Nadsonia_fulvescens_var._elongata_DSM_6958
RDW25834.1_Saccharomycetales_Dipodascaceae_Yarrowia_Yarrowia_lipolytica
XP_018737583.1_Saccharomycetales_Trichomonascaceae_Sugiyamaella_Sugiyamaella_lignohabitans
CDO52471.1_Saccharomycetales_Dipodascaceae_Geotrichum_Geotrichum_candidum
CDO54999.1_Saccharomycetales_Dipodascaceae_Geotrichum_Geotrichum_candidum

Cat8_Saccharomycetaceae_and_Saccharomycodaceae_clade

SSD60226.1_Saccharomycetales_Saccharomycodaceae_Saccharomycodes_Saccharomycodes_ludwigii
SGZ41833.1_Saccharomycetales_Saccharomycodaceae_Hanseniaspora_Hanseniaspora_guilliermondii
OBA25299.1_Saccharomycetales_Saccharomycodaceae_Hanseniaspora_Hanseniaspora_valbyensis_NRRL_Y-1626
XP_003675398.1_Saccharomycetales_Saccharomycetaceae_Naumovozyma_Naumovozyma_castellii_CBS_4309
XP_003672473.1_Saccharomycetales_Saccharomycetaceae_Naumovozyma_Naumovozyma_dairenensis_CBS_421
XP_022466353.1_Saccharomycetales_Saccharomycetaceae_Kazachstania_Kazachstania_naganishii_CBS_8797
XP_003955826.1_Saccharomycetales_Saccharomycetaceae_Kazachstania_Kazachstania_africana_CBS_2517
SMN22030.1_Saccharomycetales_Saccharomycetaceae_Kazachstania_Kazachstania_saulgeensis
XP_449478.1_Saccharomycetales_Saccharomycetaceae_Nakaseomyces_Candida_glabrata
XP_018220327.1_Saccharomycetales_Saccharomycetaceae_Saccharomyces_Saccharomyces_eubayanus
EHN00712.1_Saccharomycetales_Saccharomycetaceae_Saccharomyces_Saccharomyces_cerevisiae_x_Saccharomyces_kudriavzevii_VIN7
EJT42916.1_Saccharomycetales_Saccharomycetaceae_Saccharomyces_Saccharomyces_kudriavzevii_IFO_1802
EWH16862.1_Saccharomycetales_Saccharomycetaceae_Saccharomyces_Saccharomyces_cerevisiae_P283
Cat8_CAA55139.1_Saccharomycetales_Saccharomycetaceae_Saccharomyces_Saccharomyces_cerevisiae
AJS92705.1_Saccharomycetales_Saccharomycetaceae_Saccharomyces_Saccharomyces_cerevisiae_YJM1418
AJS81799.1_Saccharomycetales_Saccharomycetaceae_Saccharomyces_Saccharomyces_cerevisiae_YJM1273
XP_003679393.1_Saccharomycetales_Saccharomycetaceae_Torulaspora_Torulaspora_delbrueckii
GAV55967.1_Saccharomycetales_Saccharomycetaceae_Zygosaccharomyces_Zygosaccharomyces_rouxii
XP_002498603.1_Saccharomycetales_Saccharomycetaceae_Zygosaccharomyces_Zygosaccharomyces_rouxii
AQZ14297.1_Saccharomycetales_Saccharomycetaceae_Zygosaccharomyces_Zygosaccharomyces_parabailii

CDH08542.1_Saccharomycetales_Saccharomycetaceae_Zygosaccharomyces_Zygosaccharomyces_bailii_ISA1307
AQZ10426.1_Saccharomycetales_Saccharomycetaceae_Zygosaccharomyces_Zygosaccharomyces_parabailii
BAP73751.1_Saccharomycetales_Saccharomycetaceae_Kluyveromyces_Kluyveromyces_marxianus
Cat8_XP_453133.1_Saccharomycetales_Saccharomycetaceae_Kluyveromyces_Kluyveromyces_lactis
CDO93457.1_Saccharomycetales_Saccharomycetaceae_Kluyveromyces_Kluyveromyces_dobzhanskii_CBS_2104
XP_017987283.1_Saccharomycetales_Saccharomycetaceae_Eremothecium_Eremothecium_sinecaudum
XP_003646462.1_Saccharomycetales_Saccharomycetaceae_Eremothecium_Eremothecium_cymbalariae_DBVPG7215
NP_982826.2_Saccharomycetales_Saccharomycetaceae_Eremothecium_Eremothecium_gossypii_ATCC_10895
AGO10335.1_Saccharomycetales_Saccharomycetaceae_Saccharomycetaceae_sp._Ashbya_aceri
SCW03288.1_Saccharomycetales_Saccharomycetaceae_Lachancea_Lachancea_fermentati
SCU99863.1_Saccharomycetales_Saccharomycetaceae_Lachancea_Lachancea_mirantina
XP_002552389.1_Saccharomycetales_Saccharomycetaceae_Lachancea_Lachancea_thermotolerans_CBS_6340
CUS21419.1_Saccharomycetales_Saccharomycetaceae_Lachancea_Lachancea_quebecensis
SCV01935.1_Saccharomycetales_Saccharomycetaceae_Lachancea_Lachancea_nothofagi_CBS_11611
SCU97224.1_Saccharomycetales_Saccharomycetaceae_Lachancea_Lachancea_dasiensis_CBS_10888
SCV00984.1_Saccharomycetales_Saccharomycetaceae_Lachancea_Lachancea_meyersii_CBS_8951
XP_022627556.1_Saccharomycetales_Saccharomycetaceae_Lachancea_Lachancea_lanzarotensis
SCU89845.1_Saccharomycetales_Saccharomycetaceae_Lachancea_Lachancea_sp._CBS_6924

Cat8_Single-sequence_Debaryomycetaceae_Babjeviella_inositovora (no clade)

XP_018983956.1_Saccharomycetales_Debaryomycetaceae_Babjeviella_Babjeviella_inositovora_NRRL_Y-12698

Cat8_Phaffomycetaceae_and_Pichiaceae_clade

XP_020044763.1_Saccharomycetales_Ascoideaceae_Ascoidea_Ascoidea_rubescens_DSM_1968
XP_019041802.1_Saccharomycetales_Phaffomycetaceae_Wickerhamomyces_Wickerhamomyces_anomalus_NRRL_Y-366-8
XP_011271777.1_Saccharomycetales_Phaffomycetaceae_Wickerhamomyces_Wickerhamomyces_ciferrii
XP_020071922.1_Saccharomycetales_Phaffomycetaceae_Cyberlindnera_Cyberlindnera_jadinii_NRRL_Y-1542
CEP21463.1_Saccharomycetales_Phaffomycetaceae_Cyberlindnera_Cyberlindnera_jadinii
CDR37533.1_Saccharomycetales_Phaffomycetaceae_Cyberlindnera_Cyberlindnera_fabianii
ONH69489.1_Saccharomycetales_Phaffomycetaceae_Cyberlindnera_Cyberlindnera_fabianii
ODV94686.1_Saccharomycetales_Saccharomycetaceae_Pachysolen_Pachysolen_tannophilus_NRRL_Y-2460
Cat8_1_XP_002491690.1_Saccharomycetales_Phaffomycetaceae_Komagataella_Komagataella_phaffii_GS115
ANZ75826.1_Saccharomycetales_Phaffomycetaceae_Komagataella_Komagataella_pastoris
XP_022460283.1_Saccharomycetales_Kuraishia_Kuraishia_capsulata_CBS_1993
OWB67751.1_Saccharomycetales_Pichiaceae_Ogataea_Candida_boidinii
OWB86927.1_Saccharomycetales_Pichiaceae_Ogataea_Candida_boidinii
OUM55387.1_Saccharomycetales_Pichiaceae_Ogataea_Candida_boidinii
OWB81137.1_Saccharomycetales_Pichiaceae_Ogataea_Candida_boidinii
OWB63700.1_Saccharomycetales_Pichiaceae_Ogataea_Candida_boidinii
OWB74921.1_Saccharomycetales_Pichiaceae_Ogataea_Candida_boidinii
OWB59026.1_Saccharomycetales_Pichiaceae_Ogataea_Candida_boidinii
ODV82578.1_Saccharomycetales_Pichiaceae_Ogataea_Candida_arabinofermentans_NRRL_YB-2248
Cat8_XP_018209149.1_Saccharomycetales_Pichiaceae_Ogataea_Ogataea_polymorpha
XP_013936787.1_Saccharomycetales_Pichiaceae_Ogataea_Ogataea_parapolymorpha_DL-1
VEU21323.1_Saccharomycetales_Pichiaceae_Brettanomyces_Brettanomyces_naardenensis
GAV29036.1_Saccharomycetales_Pichiaceae_Pichia_Pichia_membranifaciens
XP_019016004.1_Saccharomycetales_Pichiaceae_Pichia_Pichia_membranifaciens_NRRL_Y-2026
XP_020542872.1_Saccharomycetales_Pichiaceae_Pichia_Pichia_kudriavzevii
AWU74705.1_Saccharomycetales_Pichiaceae_Pichia_Pichia_kudriavzevii
OUT20191.1_Saccharomycetales_Pichiaceae_Pichia_Pichia_kudriavzevii

Cat8_Debaryomycetaceae_and_Metschnikowiaceae_clade

RLV87758.1_Saccharomycetales_Debaryomycetaceae_Meyerozyma_Meyerozyma_sp._JA9
XP_001485621.1_Saccharomycetales_Debaryomycetaceae_Meyerozyma_Meyerozyma_guilliermondii_ATCC_6260
XP_006683732.1_Saccharomycetales_Debaryomycetaceae_Yamadazyma_Yamadazyma_tenuis_ATCC_10573
XP_002617846.1_Saccharomycetales_Metschnikowiaceae_Clavispora_Clavispora_lusitaniae_ATCC_42720
RKP29934.1_Saccharomycetales_Metschnikowiaceae_Metschnikowia_Metschnikowia_bicuspidata
XP_018713241.1_Saccharomycetales_Metschnikowiaceae_Metschnikowia_Metschnikowia_bicuspidata_var._bicuspidata_NRRL_YB-4993
SGZ53145.1_Saccharomycetales_Metschnikowiaceae_Clavispora_Candida_intermedia
SGZ58541.1_Saccharomycetales_Metschnikowiaceae_Clavispora_Candida_intermedia
PSK79111.1_Saccharomycetales_Metschnikowiaceae_Clavispora_Candida_auris
XP_025341395.1_Saccharomycetales_Metschnikowiaceae_Clavispora_Candida_haemulonis
XP_024715689.1_Saccharomycetales_Metschnikowiaceae_Clavispora_Candida_pseudohaemulonis
XP_025338733.1_Saccharomycetales_Metschnikowiaceae_Clavispora_Candida_duobushaemulonis
XP_020075091.1_Saccharomycetales_Debaryomycetaceae_Hyphopichia_Hyphopichia_burtonii_NRRL_Y-1933
XP_460549.2_Saccharomycetales_Debaryomycetaceae_Debaryomyces_Debaryomyces_hansenii_CBS767
XP_015467971.1_Saccharomycetales_Debaryomycetaceae_Debaryomyces_Debaryomyces_fabryi
CCE83004.1_Saccharomycetales_Debaryomycetaceae_Millerozyma_Millerozyma_farinosa_CBS_7064
CCE82081.1_Saccharomycetales_Debaryomycetaceae_Millerozyma_Millerozyma_farinosa_CBS_7064
XP_020066064.1_Saccharomycetales_Debaryomycetaceae_Suhomyces_Suhomyces_tanzawaensis_NRRL_Y-17324
XP_001383208.2_Saccharomycetales_Debaryomycetaceae_Scheffersomyces_Scheffersomyces_stipitis_CBS_6054
RLV93997.1_Saccharomycetales_Debaryomycetaceae_Spathaspora_Spathaspora_sp._JA1
XP_001528147.1_Saccharomycetales_Debaryomycetaceae_Lodderomyces_Lodderomyces_elongisporus_NRRL_YB-4239
CCE43097.1_Saccharomycetales_Debaryomycetaceae_Candida_Candida_parapsilosis
XP_003866499.1_Saccharomycetales_Debaryomycetaceae_Candida_Candida_orthopsilosis_Co_90-125
XP_002417419.1_Saccharomycetales_Debaryomycetaceae_Candida_Candida_dubliniensis_CD36
KGR14049.1_Saccharomycetales_Debaryomycetaceae_Candida_Candida_albicans_P57072
EMG49803.1_Saccharomycetales_Debaryomycetaceae_Candida_Candida_maltosa_Xu316
XP_026596208.1_Saccharomycetales_Debaryomycetaceae_Candida_Candida_viswanathii
XP_026593335.1_Saccharomycetales_Debaryomycetaceae_Candida_Candida_viswanathii

Supplementary material 2: List of all predicted TFBS in the regions 1000 bps upstream of *CAT8*/*CAT8-1* and *SIP4*/*CAT8-2* in *S. cerevisiae*, *K. lactis* and *K. phaffii*. TFBS were predicted by yeastract-plus.org. TFBS common in all three species are underlined.

***CAT8* / *CAT8-1* promoter**			***SIP4* / *CAT8-2* promoter**		
K. phaffii	***S. cerevisiae***	***K. lactis***	***K. phaffii***	***S. cerevisiae***	***K. lactis***
Abf1p	Abf1p	Abf1p	Abf1	Abf1	Adr1
Adr1p	Adr1p	Adr1p	Ace2	Aft1	Ash1
Arg81p	Ash1p	Ash1p	Aft1	Aft2	Bas1
Ash1p	Azf1p	Azf1p	Aft2	Arg81	**Cat8**
Bas1p	Com2p	Cbf1p	Ash1	Ash1	Cbf1
Cup2p	Crz1p	Fkh1p	Azf1	Azf1	Com2
Fkh1p	Cup2p	Fkh2p	Bas1	Bas1	Cst6
Fkh2p	Fkh1p	Gcn4p	Com2	**Cat8**	Cup2
Gcn4p	Fkh2p	Gcr1p	Crz1	Fkh1	Fkh1
Gcr1p	Gis1p	Gln3p	Fkh1	Fkh2	Fkh2
Gln3p	Haa1p	Haa1p	Fkh2	Gcn4	Gcn4
Hac1p	Hap2p	Hsf1p	Gcn4	Gcr1	Gcr1
Hsf1p	Hap3p	Mal63p	Gcr1	Gln3p	Gis1
Mal63p	Hap4p	Met4p	Gis1	Gsm1	Gsm1
**Mig1p**	Hap5p	**Mig1p**	Gln3	Mac1	Haa1
Mot2p	Hsf1p	Mot2p	Haa1	Mal63	Hac1
Mot3p	Ime1p	Mot3p	Hac1	Mcm1	Hap1-5
Ndt80p	Mcm1p	Ndt80p	Mal63	Mot2	Hot1
Nrg1p	**Mig1p**	Nrg1p	**Mig1**	Mot3	Hsf1
Pho2p	**Mig2p**	Oaf1p	**Mig2**	Ndt80	Ino2
Pip2p	Mot2p	Pho2p	Mot2	Nrg1	Ino4
Rgt1p	Mot3p	Pho4p	Mot3	Pho2	Mal63
Rlm1p	Msn2p	Pip2p	Msn2	Pip2	Mbp1
Rox1p	Msn4p	Rgt1p	Msn4	Rgt1	Met4
Rtg1p	Ndt80p	Rox1p	Nrg1	Rlm1	**Mig1**
Rtg3p	Nrg1p	Rtg1p	Pho2p	Rox1	Mot2
Stb4p	Pho2p	Rtg3p	Pip2	Rtg1	Mot3
Stb5p	Reb1p	Skn7p	Rgt1	Rtg3	Msn2
Ste12p	Rgt1p	Stb5p	Rim101	**Sip4**	Msn4
Sum1p	Rox1p	Stp1p	Rlm1	Stb5	Ndt80
Tec1p	Rph1p	Stp2p	Rox1	Ste12	Nrg1
Xbp1p	Rtg1p	Tye7p	Rph1	Tec1	Oaf1
Yap1p	Rtg3p	Xbp1p	Rtg1	War1	Pho2
	Stb5p	Yap1p	Rtg3	Xbp1	Pho4
	Stp1p	Zap1p	Stb5	Yap1	Pip2
	Stp2p		Ste12	Yrr1	Rgt1
	Sum1p		Stp2		Rim101
	Tec1p		Swi5		Rox1
	Upc2p		Tec1		Rph1
	Usv1p		Usv1		Rtg1

Yap1p	Xbp1	Rtg3
Zap1p	Yap1	Sfl1
	Yrr1	**Sip4**
		Stb5
		Sum1
		Tec1
		Tye7
		Usv1
		Xbp1
		Yap1
		Yap3
		Yrr1
		Zap1
		Znf1
